# The impact of atypical sensory processing on social impairments in autism spectrum disorder

**DOI:** 10.1016/j.dcn.2017.04.010

**Published:** 2017-05-17

**Authors:** Melissa D. Thye, Haley M. Bednarz, Abbey J. Herringshaw, Emma B. Sartin, Rajesh K. Kana

**Affiliations:** Department of Psychology, University of Alabama at Birmingham, Birmingham, AL 35233, United States

**Keywords:** Autism spectrum disorder, Sensory, Social cognition, Sensory sensitivity

## Abstract

Altered sensory processing has been an important feature of the clinical descriptions of autism spectrum disorder (ASD). There is evidence that sensory dysregulation arises early in the progression of ASD and impacts social functioning. This paper reviews behavioral and neurobiological evidence that describes how sensory deficits across multiple modalities (vision, hearing, touch, olfaction, gustation, and multisensory integration) could impact social functions in ASD. Theoretical models of ASD and their implications for the relationship between sensory and social functioning are discussed. Furthermore, neural differences in anatomy, function, and connectivity of different regions underlying sensory and social processing are also discussed. We conclude that there are multiple mechanisms through which early sensory dysregulation in ASD could cascade into social deficits across development. Future research is needed to clarify these mechanisms, and specific focus should be given to distinguish between deficits in primary sensory processing and altered top-down attentional and cognitive processes.

## 1 Introduction

Autism Spectrum Disorder (ASD) is a complex neurodevelopmental disorder characterized by deficits in social communication and the presence of restricted, repetitive behaviors (RRB) (American Psychiatric Association, 2013); current estimates state that it affects 1 in 68 children (Christensen et al., 2016). Despite the first scientific report of ASD mentioning altered sensory perception as a characteristic feature (Kanner, 1943), ASD research has historically been heavily focused on social impairments (see Leekam, 2016 for a review), with many popular theories construing it as a social disorder (including social motivation hypothesis, Dawson et al., 2005; and the mindblindness account, Baron-Cohen et al., 1985, Baron-Cohen, 1990). In recent years, however, research focusing on the sensory domain has found that sensory processing abnormalities in ASD (see Baum et al., 2015; or Marco et al., 2011; for reviews) are reported across all ages and levels of symptom severity (Leekam et al., 2007) and adversely affect both daily functioning (Suarez, 2012) and academic performance (Howe and Stagg, 2016). Such abnormalities have been documented across all sensory modalities (e.g., Kientz and Dunn, 1997), and up to 95% of parents of children with ASD report some atypical sensory behavior in their child (e.g., seeming indifference to pain, avoidance of certain sounds or textures, unusual smelling of objects, seeking out visual experiences of lights or movement; Rogers and Ozonoff, 2005). Acknowledging this, the most recent edition of the Diagnostic and Statistical Manual of Mental Disorders (DSM-5; American Psychiatric Association, 2013) lists “hyper-or–hypo-reactivity to sensory input or unusual interests in sensory aspects of the environment” as a type of restricted and repetitive behavior. Thus, there is behavioral, neurophysiological and anecdotal evidence of sensory impairment as a prevalent characteristic feature of ASD.

While prior research has often focused on the sensory and social features of ASD independently of one another, new theoretical and empirical evidence suggests a stronger relationship between the two than previously thought (Ronconi et al., 2016). Sensory and social behaviors may arise from a common underlying mechanism and/or may exert reciprocal influence on each other in the course of a child’s development (Gliga et al., 2014). This relationship is also evident from findings of early abnormal sensory sensitivity to stimuli predicting later joint attention and language development (Baranek et al., 2013), development of social play (Miller Kuhaneck and Britner, 2013), increased withdrawal and negative temperament (Brock et al., 2012), and higher levels of social impairment in adults with ASD (Hilton et al., 2010). Thus, the relationship between social and sensory features in ASD may be bidirectional and inter-dependent. For example, a child that is overly sensitive to loud noises may withdraw from socio-communicative environments that are over-stimulating, leading to less practice with social scenarios and ultimately resulting in a breakdown of successful social interaction.

This review will examine the behavioral and neurobiological studies on social and sensory processing in ASD to explore the relationship between sensory and social impairments in ASD. Specifically, we will 1) discuss the possible mechanisms by which atypical sensory processing across the five basic senses could manifest in the social deficits characteristic of individuals with ASD; 2) review the existing hypotheses that have attempted to integrate these features; and 3) review evidence from neuroimaging studies to highlight differences in sensory and social representations often observed in ASD. We will also discuss how early abnormalities in sensory processing can be compounded over time, creating a maladaptive developmental trajectory of cascading delays and deficits.

The relationship between sensory and social processing may occur at many hierarchical levels. At the very basic level, sensory receptors are stimulated by environmental stimuli. This sensory information is then relayed to the brain to create a subjective neural representation, a process known as perception (Perrone, 2007). Sensation and perception are inter-related constructs. A breakdown of sensation results in a lack of perception, and similarly, without perception the activation of a sensory receptor is meaningless (Goldstein and Brockmole, 2017). Often in research, perception is the behavioral output of interest, which presumes sensation. A related confound in sensory research is attention. Specifically, an individual may sense and perceive a stimulus, but can fail to attend to it in an expected way (Heald and Nusbaum, 2014, Näätänen et al., 1990). It can, thus, become problematic to disentangle these constructs and know definitively if a sensory, perceptual, or attentional deficit is underlying a given response. Thus, an atypical behavioral or neural outcome can reflect a breakdown at any point within this adopted hierarchy. In many cases, the research has not progressed enough to allow for a definitive disentangling of these concepts, but evidence of sensory, perceptual, and attentional abnormalities in ASD individuals will be outlined where possible. The study of these processes in each sensory modality and their role in social functioning in individual with ASD will be the primary focus of this paper.

## 2 Vision

Individuals on the autism spectrum often seek out or avoid intense visual stimulation (Leekam et al., 2007). Atypical visual processing has been widely documented in individuals with ASD (see Simmons et al., 2009 for a review), with alterations in basic perceptual functions, including contrast sensitivity (Behrmann et al., 2006, Bertone et al., 2003), boundary detection (Vandenbroucke et al., 2008), field of view size (Song et al., 2015) and color perception (Franklin et al., 2008). Deficits in visual form processing (Spencer and O'Brien, 2006) and motion perception (see Kaiser and Shiffrar, 2009 for a review), reduced susceptibility to illusion (Happe, 1996, Bolte et al., 2007), superior visual search (Jolliffe and Baron-Cohen, 1997), a local processing bias (Dakin and Frith, 2005), spatial attention impairments (Haist et al., 2005), and altered oculomotor function (Goldberg et al., 2002) are also reported. Behaviorally, some differences manifest as enhanced perceptual abilities in ASD, particularly in basic low level visual search tasks; conversely, these may be disruptive to efficient processing in fast-paced, complex visual environments (Happe and Frith, 2006, Mottron et al., 2006, Pellicano and Burr, 2012). Differences in vision have also emerged as one of the earliest stable markers of ASD, with saccade duration in 7-month olds reliably distinguishing children later diagnosed with ASD (Wass et al., 2015). These differences may play a role in social impairments in ASD, as perception of social cues drives visual attention patterns, and thus is crucial in social development and interpersonal interactions. Infants’ preferential attention to eyes and faces (Maurer and Salapatek, 1976) reflects how early social understanding is built on observation. The frequency of lateral glances and visual hypo-responsivity predict poorer social skills and greater overall ASD symptomology (Hellendoorn et al., 2014, Kern et al., 2007). Further, children with visual impairments often have social deficits due to difficulties in social learning through visual cues, modeling, or feedback (Kekelis, 1992, McGaha and Farran, 2001). In the subsections below, we discuss the relevance of vision in important social functions widely studied in autism (eye gaze, face processing, and biological motion) and the developmental impact of early visual processing abnormalities.

### 2.1 Gaze processing

Previous research has questioned whether atypical visual processing underlies poor eye contact and joint attention, impairments of both are reported in ASD (Sigman et al., 1986, Charman et al., 1997, Stone et al., 1994, Leekam et al., 1997). Joint attention (JA) involves two participants coordinating mutual engagement with their mutual focus on a third entity (Tomasello, 1995). Developmentally, eye contact serves an important early social function for infants (Stern, 1985), regulating face-to-face interactions (Lee et al., 1998, Leekam et al., 1997) and fostering emerging social skills. JA is also critical to social and cognitive development, predicting language abilities (e.g., Gillespie-Lynch et al., 2015), understanding intention (Mundy and Newell, 2007), and pretend play (Rutherford et al., 2007b). Eye contact and JA are dependent upon an intact ability to detect gaze and understand gaze cues. Many studies suggest that individuals with ASD are able to determine the direction of others’ gaze, but fail to effectively use this information socially (Leekam et al., 1997, Pelphrey and Carter, 2008). However, others have found ASD individuals to be less accurate in judging the intent behind both direct and averted gaze, particularly in more ambiguous situations (Senju et al., 2003, Ashwin et al., 2009, Forgeot d'Arc et al., 2016). At the neurobiological level, a failure to shift from the magnocellular to parvocellular pathway in ASD early in life (Mundy et al., 2009, McCleery et al., 2007) could underlie delayed gaze detection abilities, since these pathways coordinate distinct patterns of visual preference. Additionally, poor gaze detection during JA has been associated with decreased occipital pole activation in ASD participants (Tanabe et al., 2012). Engagement in JA also involves extending gaze detection to gaze following, requiring the regulation of visual attention. Evidence for difficulties in oculomotor control and visual attention regulation are more robust in ASD. An inability to visually disengage from a central to a peripheral stimulus at 14-months was predictive of later ASD diagnosis in high-risk infants (Elsabbagh et al., 2013a), and poor visual disengagement is seen in ASD across various tasks and ages (see Sacrey et al., 2014). Additional elements of oculomotor control, such as smooth pursuit eye movements are also disrupted in ASD (Takarae et al., 2004), possibly related to reduced functional connectivity between V1 and inferior frontal areas (Villalobos et al., 2005). Thus, difficulties perceiving gaze cues may contribute to joint attention difficulties in individuals with ASD.

### 2.2 Face processing

Intact visual processing is a prerequisite for attending to and recognizing faces, making face processing a social as well as perceptual skill. Face processing abnormalities in individuals with ASD, including less focus on eyes and increased looking to the mouth (Klin et al., 2002), have been studied extensively (e.g., Schultz et al., 2000, Teunisse and Gelder, 1994, Klin et al., 1999, Pierce et al., 2001, Nomi and Uddin, 2015, Weigelt et al., 2012). In addition, poorer facial identity recognition (e.g., Kirchner et al., 2011), facial memory (e.g., Wilson et al., 2010), face discrimination (e.g., Rutherford et al., 2007a), and deficits in facial emotion recognition (e.g., Hobson, 1986, Baron-Cohen, 1991, Harms et al., 2010) have also been reported in ASD. At the neural level, individuals with ASD show hypo-activation in the fusiform face area (FFA), superior temporal sulcus (STS), and the occipital face area (Schultz et al., 2000, Humphreys et al., 2008). Atypical face processing in ASD could arise from sociocognitive factors, or reflect visual processing deficits more broadly, such as distinguishing between both social and non-social stimuli that are visually complex or highly similar, including upright faces, inverted faces, and cars (Ewing et al., 2013); and poor ability to discriminate between novel, and perceptually similar objects (Greebles) and faces (Scherf et al., 2008). Difficulties in rapidly processing visual information are also seen in ASD, leading to reduced attention to faces (Charrier et al., 2016). These studies suggest that impairments perceiving certain visual characteristics (i.e., complexity, higher frequency, higher distortion, fast-moving) contribute to face processing and emotion recognition difficulties in ASD. These basic impairments may be exacerbated by the rapid, complex visual information conveyed by human faces in social interactions, and further compounded by social-cognitive impairments characteristic of ASD.

### 2.3 Biological motion processing

According to Johansson (1973), biological motion (motion of humans and animals) is characterized by distinct, highly complex spatiotemporal movement patterns. Preferential attending to biological motion has been reported in typically developing children by 2 days of age (Simion et al., 2008). This plays an important role in developing joint attention, imitation, emotion recognition, and social cognition in general (Pavlova, 2012). However, studies have reported disruptions in attending to and in recognizing biological motion in individuals with ASD (Kaiser and Pelphrey, 2012). Studies using point-light displays, which allow motion perception without the confound of form, reveal that children with ASD fail to preferentially attend to biological over non-social motion (Klin et al., 2009, Blake et al., 2003, Annaz et al., 2012). Moreover, individuals with ASD have difficulty using information from biological motion to infer emotions (Nackaerts et al., 2012), recognize faces (O'Brien et al., 2014) and follow pointing (Swettenham et al., 2013). Some evidence suggests that motion processing impairments in ASD are specific to biological motion perception (e.g., Koldewyn et al., 2011), possibly stemming from a failure to modulate posterior STS activity specifically for biological motion (Pelphrey et al., 2007, Pelphrey and Carter, 2010). However, others have reported similar impairments in ASD in coherent motion processing more broadly (e.g., Manning et al., 2013, Freitag et al., 2008). Individuals with ASD also show reduced activity in V1 and other early visual areas during both coherent and biological motion processing (Robertson et al., 2014; Kröger et al., 2014). Further, poorer recognition of basic motion patterns in ASD has been found to be correlated with the ability to recognize emotions from biological motion (Atkinson, 2009). There is additional evidence that global motion processing abilities might depend on task speed (e.g., Manning et al., 2013) or duration (Hadad et al., 2015), raising the possibility that motion perception in ASD is technically intact, but too inefficient to process complex, fast-paced interactions in social scenarios. Thus, evidence suggests some deficits in low-level visual motion perception in ASD, stemming from early visual processing areas, which may contribute to biological motion processing impairments, which in turn is central to the social impairments seen in ASD.

### 2.4 Developmental consequences of atypical vision

Prospective studies of infants at high risk for ASD suggest atypical visual processing is present within the first year of life. Infants later diagnosed with ASD make faster saccades and have difficulty in visual disengagement at 7 months of age (Wass et al., 2015, Elsabbagh et al., 2013a). Superior visual-search abilities at 9 months were also predictive of later ASD symptoms (Gliga et al., 2015). Interestingly, such alterations in visual processing may precede quantifiable differences in social functioning. For example, gaze following is intact at 7 and 13 months in children later diagnosed with ASD (Bedford et al., 2012); these infants also attend to faces as frequently or even more frequently than typically developing children in the first year of life (Elsabbagh et al., 2013b, Jones and Klin, 2013, Yirmiya et al., 2006), decreasing this social attention thereafter and falling behind typical development in the second year (Jones and Klin, 2013, Ozonoff et al., 2010). It is possible that pre-existing visual processing deficits may disrupt developing social skills by preventing the perception of visual cues that signal social rewards, making the cause and effect of social interactions unpredictable. Over time, these infants might begin to lose interest in these “unpredictable” social interactions, and instead seek out repetitive and predictable non-social stimulation (Gliga et al., 2014). This view aligns with the findings that visual attention to social stimuli in ASD is initially increased, as increased looking time in infants is interpreted as a marker of an unexpected event (e.g., Csibra et al., 2016). Moreover, altered eye contacts and visual social attention in children with autism in early life can result in a practice effect and lead to a secondary neurological assault, ultimately resulting in a brain-behavior cycle with adverse effects on social life (Mundy and Crowson, 1997, Klin et al., 2015). Thus, atypical visual processing in early development in ASD could have cascading deleterious effects on subsequent social and cognitive development through the ongoing process of experience-dependent learning.

## 3 Auditory processing

Hearing, like vision, is an important aspect of successful participation in social-communicative interactions. The earliest exposure to auditory stimuli occurs in the intrauterine environment; and postnatal studies indicate infants’ recognition and preference for mother’s voice (Purhonen et al., 2004). Infants engage in preferential orientation to, and discrimination of speech versus non-speech sounds (see Moore and Linthicum, 2007 for review), which is predictive of both receptive language development (Paul et al., 2007) and expressive vocabulary (Vouloumanos and Curtin, 2014). Early auditory inputs facilitate the extraction of socially salient information from the environment. Thus, altered sensation, perception, and attention to different auditory stimuli may have direct implications for social functioning.

Atypical auditory processing is well-documented in ASD (see O'Connor, 2012 for review) with a profile of enhanced pitch perception (Bonnel et al., 2003, Bonnel et al., 2010, O'Riordan and Passetti, 2006), increased sensitivity to loud noises (Khalfa et al., 2004), lack of auditory orientation (Paul et al., 2007), impaired perception of prosody (Järvinen-Pasley et al., 2008), and diminished auditory stream segregation (Teder-Sälejärvi et al., 2005). Sensory level deficits within the central auditory nervous system and relevant auditory pathways in ASD have been reported using auditory brainstem response (ABR) paradigms (Kwon et al., 2007); and this delayed response distinguishes ASD from other neurodevelopmental disorders (Källstrand et al., 2010). Delayed ABR has also been seen in response to phonemes, which are the basic units that comprise language, but not to non-speech sounds such as clicking which elicited a typical brainstem response (Russo et al., 2009). These findings collectively indicate that change in pitch, accompanied by increased complexity of auditory stimuli, is an area of significant deficit in ASD. The central hub of auditory processing is the primary auditory cortex located within Heschl’s gyrus in the superior temporal cortex (Belin et al., 2004). Structural imaging studies of adults with ASD reveal increased cortical thickness in Heschl’s gyrus although these findings were accompanied by global neuroanatomical differences in ASD (Hyde et al., 2010). With competing sources of auditory information, individuals with ASD display limited ability to isolate certain features of concurrent auditory information (Lepistö et al., 2009) and are less able to focus auditory attention on the more salient information in the environment compared to controls, even after controlling for IQ and hearing ability (Teder-Sälejärvi et al., 2005). Thus, even in the absence of a basic auditory sensory impairment, some individuals with ASD have difficulty filtering auditory information which may underscore a general deficit in integrating information. This deficit in auditory stream segregation interferes with the ability to perceive or attend to social information in the presence of competing auditory input. Thus, abnormal auditory processing is consistently reported in ASD and stems from atypical sensation, altered perception, and lack of preferential attention to auditory stimuli which directly impacts successful social engagement.

### 3.1 Speech recognition

Possibly the most socially salient auditory stimuli in early development is the mother’s voice. In contrast to typically developing peers, infants with autism do not preferentially attend to mother’s speech or child-directed speech (Dawson et al., 1998, Klin, 1991, Paul et al., 2007). Infants at high-risk for ASD diagnosis respond to speech versus non-speech sounds less than low-risk infants. This trend is associated with ASD symptomatology in the high-risk group and with language development in the low-risk group (Curtin and Vouloumanos, 2013). Children with ASD who do not preferentially orient to child-directed speech have poor sound discrimination, highlighting the role of basic sensory impairment in social behavior (Kuhl et al., 2005). Children with ASD display oversensitivity to local changes in auditory information such as pitch at the expense of global auditory information such as speech (Foster et al., 2016). There are conflicting reports, however, finding both disrupted (Foxton et al., 2003) and intact (Heaton et al., 2007, Mottron et al., 2000) global auditory processing. Research utilizing a mismatch negativity (MMN) EEG paradigm found reduced preference for social and affective auditory information in children with ASD as well as decreased sensory discrimination between expected and unexpected speech (Fan and Cheng, 2014) suggesting that social information is filtered out and ignored at a basic sensory or perceptual level. Researchers have also reported delayed latencies of event-related potentials (ERPs) in ASD in response to auditory stimuli using EEG and MEG (Roberts et al., 2010, Whitehouse and Bishop, 2008), and this pattern of auditory processing is associated with language functioning (Oram Cardy et al., 2008). However, there is debate over whether speech-related auditory impairment in ASD is a sensory deficit or an orienting deficit (Ceponiene et al., 2003). Evidence supporting the role of attention deficit in auditory processing in ASD comes from fMRI studies of adults with ASD who show decreased activation in STG in response to speech stimuli, a pattern not seen in response to non-social sound stimuli (Gervais et al., 2004, Lai et al., 2011). Furthermore, adults with ASD show greater recruitment of right STG, compared to left, which was seen in typically developing participants passively listening to speech. The reduced activation in left hemispheric language processing areas might underlie a core deficit in processing speech which may inhibit the perception and production of language (Boddaert et al., 2004). Altered recruitment of STG in ASD may indicate a sensory level deficit or a breakdown of higher order functions such as attention or language processing due to the dual roles of the STG in both language and auditory processing (see Redcay, 2008 for review). There is additional evidence supporting the role of attention in auditory discrimination tasks (Dunn et al., 2008). However, it should be noted that high functioning individuals with ASD score in the atypical range on sensory processing behavioral measures and these scores predict the severity of social functioning (Hilton et al., 2010). Thus, failure to preferentially respond to speech early in development may be indicative of a lower level sensory or perceptual deficit or a higher order impairment in attention or language processing which can cascade into impairments in speech recognition.

Enhanced pitch discrimination, well-documented in autism (Bonnel et al., 2003, Bonnel et al., 2010, Bouvet et al., 2014, O'Riordan and Passetti, 2006), extends to both music (Mottron et al., 2000) and speech (Järvinen-Pasley and Heaton, 2007) and represents a relative area of strength in auditory processing in autism (see O'Connor, 2012 for review). It should be noted that language delays are reported among adults with ASD who display enhanced pitch perception (Bonnel et al., 2010, Eigsti and Fein, 2013, Jones et al., 2009). In addition, there is neural evidence of enhanced pitch processing of non-speech sounds, but not speech sounds in ASD (Yu et al., 2015) possibly suggesting that superior pitch processing in childhood may lead to attentional exclusion of language-related pitch and subsequent delays in language acquisition. For instance, a child with superior pitch perception might be more attuned to all changes in pitch in the environment without selectively attending to speech-related changes in pitch. Enhanced auditory processing of pitch and oversensitivity to loudness (Khalfa et al., 2004) can result in heightened awareness of simple perceptual features of auditory information at the exclusion of complex auditory input such as speech (Lin et al., 2016) and the inability to discriminate the salient social information in the auditory environment, an ability known as auditory stream segregation. Currently, the relationship between pitch perception and language ability or age is not explicitly clear. Nevertheless, these factors are indicative of an early maladaptive auditory processing profile in children with autism.

### 3.2 Prosody and evaluation of affect

Prosody is an expressive mechanism of speech that allows for nuanced exchange of emotion and intention in communication (Frühholz et al., 2012). Inappropriate use of prosody (Kanner, 1943, Järvinen-Pasley et al., 2008, Wang et al., 2006) as well as difficulty extracting socially salient features from speech such as vocal affect (Järvinen-Pasley et al., 2008) is associated with worse social and communication skills (Paul et al., 2005) and is consistently reported in ASD. ABR research suggests that changes in pitch that are inherent to affective prosody do not evoke a rapid brainstem response in ASD participants which has ramifications for decoding pitch in affective language (Russo et al., 2008). Although not a basic sensory mechanism, the perception of and attention to emotionally-laden speech has implications for social functioning. Prosodic differences in ASD may suggest a breakdown of higher-order perceptual or attentional processes. Additionally, altered sensory response to auditory stimuli coupled with impaired integration of perceptual information may have cascading effects on the use and understanding of language. Individuals with ASD may have a deficit specific to affective prosody. This is supported by findings of unimpaired perception of pragmatic prosody, but disrupted perception of affective prosody despite intact pitch discrimination and direction recognition in ASD (Globerson et al., 2015). Neuroimaging studies of affective prosody reveal that, in addition to IFG and STG activation seen in the control group, participants with ASD recruit the right caudate nucleus and rate the stimuli as less emotionally intense (Gebauer et al., 2014). Furthermore, more widespread activation of IFG and pSTS is also noted in the ASD group compared to controls in response to angry prosody perhaps indicating a lack of habitual processing or expertise in perception of affective prosody (Eigsti et al., 2012). MEG studies have found longer response latencies and reduced recruitment of the left hemisphere in individuals with ASD in response to rapidly presented auditory stimuli. Additionally, this pattern of response was associated with vocal affect recognition indicating that impairments in affect recognition impact rapid processing of socially salient auditory information (Demopoulos et al., 2015). Further evidence of altered neural responses to prosody comes from studies of irony (Wang et al., 2006), prosody perception (Hesling et al., 2010), and word-level affective prosody (Korpilahti et al., 2007). Therefore, problems in evaluating affect from auditory stimuli in individuals with ASD can directly impact social reciprocity by limiting their ability to extract socially relevant information from voices and respond appropriately. Although it is evident that many individuals with ASD experience basic sensory impairments, there are also important perceptual and attentional mechanisms that lead to problems in social behavior. It is therefore likely that atypical sensation, altered perception, and inattention occur at varying levels across different auditory paradigms and social settings.

### 3.3 Developmental considerations

Current evidence suggests that atypical auditory processing in ASD occurs early in development. Certain auditory profiles (enhanced pitch perception and discrimination) are seen predominantly in children with ASD and to a lesser degree in adults with ASD. Enhanced pitch perception is only reported in a subset of adults with ASD who predominantly have language delays (Bonnel et al., 2010, Eigsti and Fein, 2013, Jones et al., 2009). Conversely, the ability to process rapidly presented speech decreases with age in both typical development and in ASD; however, the decline in processing begins earlier in ASD (Mayer and Heaton, 2014). This has direct implications for social functioning as the interpersonal socio-communicative world is fast and dynamic. Collectively, these areas of atypical auditory processing in ASD suggest an auditory profile of oversensitivity to basic auditory features at the expense of the ability to filter out background noise and selectively attend to speech or other relevant social cues. Additionally, abnormal perception of affective prosody has direct implications for decoding intentions and reciprocating social exchanges. Thus, atypical auditory processing in ASD is noted early in development and has cascading effects on speech processing, social engagement, and language acquisition.

## 4 Tactile processing

Touch is considered one of the most basic ways in which individuals interact with the world around them (Barnett, 1972). Touch plays a significant role in communication (Hertenstein, 2002, Hertenstein et al., 2006, Langland and Panicucci, 1982), developing social bonds (Dunbar, 2010, Langland and Panicucci, 1982), and overall physical development (Field, 1998, Polan and Ward, 1994). Recent findings suggest that touch also promotes the development and connectivity of brain areas (Brauer et al., 2016; Björnsdotter et al., 2014) associated with social cognition and the “social brain” (Adolphs, 2009, Brothers, 2002, Frith, 2007). Stimulation of C-tactile (CT) afferents, nerve fibers that process affective and limbic touch (Wessberg and Norrsell, 1993), have been shown to correlate with activation of regions of the social brain (Kaiser et al., 2016, Gordon et al., 2013, Björnsdotter and Olausson, 2011, Björnsdotter et al., 2009, Olausson et al., 2002, Olausson et al., 2010), supporting the hypothesis that skin is a “social organ” (Olausson et al., 2002, Kaiser et al., 2016, McGlone et al., 2014, Löken and Olausson, 2010). Recent evidence supports both hypo-and-hyper-reactivity to tactile stimuli in ASD, with these responses varying according to stimuli and context (Lane et al., 2011, Crane et al., 2009, Ben-Sasson et al., 2007, Tomchek and Dunn, 2007, Allely, 2013, Brown and Dunn, 2010, Cascio et al., 2008). Individuals with ASD display abnormal detection of tactile stimuli (Blakemore et al., 2006) as well as a lack of habituation to tactile stimuli (Tannan et al., 2008). Mechanistically, some have suggested that alterations in GABAergic feedforward loops might play a role in atypical tactile responsivity in ASD (e.g., Tannan et al., 2008, Puts et al., 2014, Tavassoli et al., 2016). Studies also suggest an abnormal functioning (hypo: Kaiser et al., 2016; hyper: Riquelme et al., 2016) and abnormal numbers (less: Silva and Schalock, 2016) of CT afferents in ASD populations. Thus, although individuals with ASD most likely have an altered experience of touch and pain, it is not likely that they always exhibit hypo or hyper reactivity.

### 4.1 Tactile processing and its role in social functioning in ASD

There is evidence to suggest that irregularities in touch and tactile perception may be associated with broad levels of social dysfunction in ASD. For example, touch-seeking behaviors have been found to predict levels of social impairment, and tactile hyporesponsivity was associated with both poorer social functioning and nonverbal communication skills (Foss-Feig et al., 2012). Differences in tactile processing and tactile preference behaviors in ASD are observed in early infancy (Mammen et al., 2015). Further, several studies suggest that maternal touch in early infancy critically influences a secure attachment later (Weiss et al., 2000). Social touch has been found to increase self-esteem, well-being, health status, life satisfaction and self-actualization, faith or belief, and self-responsibility (Butts, 2001), while a lack of social touch can lead to higher levels of anxiety, stress, and depression (Gupta et al., 1998, Hertenstein, 2002, Weiss et al., 2001), which are commonly seen in ASD population (Wallace et al., 2016, Uljarević et al., 2016, Kerns and Kendall, 2012, Ghaziuddin et al., 2002). Atypical touch during infancy can develop into critical deficits later in life, specifically in regards to attachment. While individuals with ASD are capable of forming a secure attachment to their caregivers (Shapiro et al., 1987, Teague et al., 2017), they tend to be less securely attached than their typically developing peers (for a *meta*-analysis, see Rutgers et al., 2004). Further, individuals with ASD who have secure attachments tend to have less socially severe symptoms than individuals with ASD who are not securely attached, suggesting symptom severity and overall level of functioning could impact the strength of attachment (Capps et al., 1994).Touch is important in developing attachment during infancy through both maternal stimulation and orienting. Infants who are later diagnosed with ASD have been observed to have less maternal touch stimulation (Baranek, 1999), and failure to orient has been associated with poor attachment (Reece et al., 2016, Weiss et al., 2000). Therefore, a lack of social touch early in development can have important social and interpersonal implications.

Touch is also important in developing social bonding. Positive tactile stimuli (touch, warmth, odors) can release oxytocin (Uvnäs-Moberg, 1998), the neuropeptide primarily involved in social bonding. Interestingly, oxytocin has been found to increase the perceived pleasantness of the touch of opposite gender, along with activity in parts of the social brain (Scheele et al., 2014). However, the behavioral and neural effects of oxytocin were negatively correlated with autistic-like traits, suggesting these effects to be blunted in individuals with autistic-traits. This may lead to a limited seeking of touch in the interpersonal interactions of individuals with ASD. Further evidence of attenuation of oxytocin in ASD comes from abnormalities in oxytocin peptide and plasma levels (Green et al., 2001, Modahl et al., 1998), and alterations in: the gene that encodes the oxytocin receptor, OXTR, (Ebstein et al., 2009, Hammock and Levitt, 2006), in oxytocin receptors (Campbell et al., 2011, Liu et al., 2010, Skuse et al., 2014, Wermter et al., 2010, Wu et al., 2005), and in epigenetic mechanisms (Gregory et al., 2009, Kumsta et al., 2015). It should be noted that the use of oxytocin in ASD individuals in clinical settings is becoming increasingly popular (for a review see, Anagnostou et al., 2014).

In addition to the basic sensory and perceptual level tactile deficits noted in ASD, failure to socially orient may also be dependent on touch. Atypical tactile perception around the face and mouth could disrupt tactile stimulation of the orienting reflex, reducing face-to-face orienting and the positive social attention associated with it (Sokolov, 1963). A recent study (Silva et al., 2015) explored this concept using the Autism Touch and Self-Regulation Checklist. In addition to finding a relationship between overall severity of sensory abnormalities and the severity of ASD symptoms, this study found that five questions related to touch/pain responses on the face and mouth correctly identified 83% of the ASD population from typically developing controls. Further, when the researchers included all questions regarding failure to orient, 91% of the ASD population was correctly identified. Future research should look further into the importance of tactile perception in orienting and self-regulation in infancy, as well as its impact on other social domains not previously explored.

## 5 Olfaction and gustation

While there is relatively less research on olfaction and gustation in ASD, there is evidence of atypical response, disrupted taste detection (Tavassoli and Baron-Cohen, 2012), in these domains. The literature available on olfaction is varied on whether ASD individuals are hypo- or hyper − sensitive, with several studies reporting intact odor detection, some finding problems with odor identification (OI; Suzuki et al., 2003, Bennetto et al., 2007), and others reporting problems with odor detection but intact OI (Dudova et al., 2011). Overall, difficulties in eating behavior and sensitivity to smell are common concerns for individuals with ASD. Youth with ASD have been found to be more selective regarding food groups, textures, tastes, and temperatures, and are more likely to exhibit higher levels of food refusal (Bennetto et al., 2012, Wiggins et al., 2009;). Individuals with ASD are also more likely to have a body mass index (BMI) within the obesity or overweight range for their ages (Bennetto et al., 2012), and their selective food preferences have been related to patterns of restricted and repetitive behaviors and olfactory functioning (Bennetto et al., 2012, Wiggins et al., 2009). Moreover, in ASD, there is a relationship between OI and ratings of initiation, maintenance, and social interchange during conversation (Bennetto et al., 2007). Brang and Ramachandran (2010) suggest that olfactory bulb dysgenesis, resulting in reduced vasopressin and oxytocin receptor binding (related to social bonding) could be one of the neural foundations of autism. Recently, Zou et al. (2016) reported a positive relationship between olfactory sensitivity and the extent of TD individuals’ social network. Interestingly, this study found that functional connectivity of the amygdala with the orbitofrontal cortex, a connection that has been suggested to impact the repetitive, stereotypical behaviors of ASD in socio-emotional cognition and behavioral self-regulation (Bachevalier and Loveland, 2006), appeared to be related to both of these factors (olfactory sensitivity and the extent of the social network). The impact of olfaction on emotion is further evident from findings of impairments in olfaction and social cognition in patients with bipolar disorder (BD; Lahera et al., 2016). In the BD population, there are relationships between OI and affect recognition and theory of mind, all of which are consistently found to be deficits in autism (Baron-Cohen, 1997, Baron-Cohen et al., 1985, Gallagher et al., 2000, Harms et al., 2010, Bölte and Poustka, 2003). These findings suggest that future studies should further examine the relationship between olfaction and the social symptoms of ASD.

## 6 Multisensory integration

The integration of multisensory stimuli is essential for the perception of complex social information. For example, social interactions require the integration of another person’s voice, face, lip movements, and gestures, failure of which may lead to misinterpretation and abnormal social response. Even if the perception of each individual sense is intact, the integration of these senses into a perceptual whole may fail (Iarocci and McDonald, 2006), and the integration affords more information than the sum of its components (Stein and Stanford, 2008). The automatic integration of multimodal stimuli creates a predictable social environment out of “noise” and inevitably influences how an individual interacts socially within that environment. Multisensory integration begins early in the stream of processing (Foxe and Schroeder, 2005) and is influenced by feed-forward operations before reaching higher-level processing centers of the brain (Stein and Stanford, 2008). Alterations in basic sensory integration have been reported in ASD (Waterhouse et al., 1996), with evidence of abnormal integration of auditory and visual stimuli during the flash-beep illusion, either perceiving the illusion over a longer temporal window (Foss-Feig et al., 2010), or being less susceptible to the illusion (Stevenson et al., 2014b). There is evidence of a relationship between ASD symptoms and a bias to perceive auditory stimuli that occur before visual stimuli as concurrent (Donohue et al., 2012). Individuals with ASD benefit less from the addition of auditory information to a visual search task (Collignon et al., 2013), show decreased multisensory facilitation to audiovisual inputs (Brandwein et al., 2013), and exhibit altered cortical recruitment during simultaneous audio-visual stimuli presentation (Russo et al., 2010). In addition, ASD individuals are less susceptible to visual-tactile illusions (Cascio et al., 2012, Greenfield et al., 2015) and struggle to integrate visual stimuli into motor planning and execution (Dowd et al., 2012). Multisensory integration has also been related to social functioning in ASD. For example, a recent study found the ERP response associated with multisensory integration to be reduced as a function of ASD symptom severity (Brandwein et al., 2015). In addition, social communication in ASD has been associated with abnormal upregulation of visual regions during auditory processing (Jao Keehn et al., 2016), indicating altered neural recruitment among the senses. Moreover, impairments in perceptual-motor integration also have been associated with communication and social deficits in ASD (Linkenauger et al., 2012). In summary, individuals with ASD show altered integration of senses across multiple domains, which may impact their social functioning.

### 6.1 Language development

The atypical language development in ASD (Tager-Flusberg et al., 2005) is an important contributor to social functioning and may be impacted by deficits in multisensory integration. In TD individuals, information about a speaker’s facial movements and gestures facilitates speech comprehension (Rosenblum, 2008, MacLeod and Summerfield, 1987, Butcher et al., 2000. Individuals with ASD often struggle to appropriately integrate additional visual information to auditory speech (Iarocci et al., 2010, Mongillo et al., 2008, Smith and Bennetto, 2007), which may impair comprehension. For example, infants at high-risk for ASD do not show differential looking during congruent and incongruent speech and lip-movement; this indicates difficulty matching auditory and visual information (Guiraud et al., 2012). Less susceptibility to the McGurk effect in children with ASD (Williams et al., 2004a, Williams et al., 2004b, Bebko et al., 2014, de Gelder et al., 1991, Stevenson et al., 2014a) (although this appears to normalize at older age ranges (Taylor et al., 2010)) may demonstrate limited influence of visual stimuli on the perceived speech phoneme (McGurk and Macdonald, 1976). There is also evidence of impaired lip-reading ability in ASD (Foxe et al., 2015), which depends on proper detection and integration of congruent audio-visual speech information (Dodd, 1979). While listening to auditory speech, individuals with ASD struggle to integrate previous exposure to speaker-specific facial information to optimize auditory speech recognition (Schelinski et al., 2014). Lastly, individuals with ASD do not benefit from the addition of gestures to auditory speech (Silverman et al., 2010) and do not properly synchronize gestures with their own speech to aid comprehension (de Marchena and Eigsti, 2010). Recruitment of regions such as the STG and STS during concurrent speech and beat gestures is found to be absent in children with ASD, with increased activity in visual areas; this was associated with increased social deficits (Hubbard et al., 2012). Altogether, there is evidence that individuals with ASD fail to integrate visual cues to speech, which may negatively impact speech comprehension, a function critical to social behavior.

### 6.2 Emotion recognition

The ability to accurately perceive and recognize emotions is essential to appropriate social functioning and involves the integration of facial expressions, vocal tone, posture, and gestures. In TD, there is evidence that visual and auditory stimuli (Massaro and Egan, 1996, Piwek et al., 2015, Stienen et al., 2011, de Gelder and Vroomen, 2000) as well as visual and olfactory stimuli (Novak et al., 2015) are integrated in emotion detection. Several studies have indicated that individuals with ASD do not appropriately integrate multisensory information in the context of emotion recognition. For example, adults with ASD struggle to discriminate whether faces and voices have congruent or incongruent emotion (O'Connor, 2007) and receive less benefit from bimodal (audio-visual) information when differentiating fear and disgust (Charbonneau et al., 2013) or identifying other emotions (Xavier et al., 2015). There is evidence of an altered temporal phase response to paired fearful faces and voices (Magnée et al., 2008), along with evidence that such altered neural responses may be modulated by attention (Magnée et al., 2011). Individuals with ASD recruit alternate brain regions in the parietofrontal network during the audio-visual integration of emotion cues, compared to controls, which recruit regions in frontal and temporal association cortices (Doyle-Thomas et al., 2013a). While not all studies have found TD-ASD differences in multimodal processing of emotion (Vannetzel et al., 2011, Magnée et al., 2007), overall, there is great evidence that deficits in multisensory integration may hinder emotion recognition in ASD.

### 6.3 Imitation

Motor imitation requires the integration of a stimulus with one’s own proprioceptive movements. The act of imitation has been proposed to provide a substrate for the development of theory of mind, empathy (Meltzoff and Decety, 2003), and peer relationships (Rubin et al., 2011). Many studies have observed imitation deficits in ASD (Williams et al., 2004a, Williams et al., 2004b, Edwards, 2014), and ASD symptom severity has been negatively associated with imitation abilities (Edwards, 2014). These deficits in imitation could potentially be influenced by weaknesses in integrating external inputs with one’s own proprioceptive behavior. The rubber hand illusion has been used as a paradigm to investigate visual-tactile integration (essential for imitation) in ASD. Among non-clinical adults, increased ASD traits have been associated with decreased susceptibility to the rubber-hand illusion (Palmer et al., 2013). Individuals with ASD also take longer to show the effects of the illusion (Cascio et al., 2012), are less sensitive to aspects of the illusion (Paton et al., 2012), and may rely more on proprioceptive inputs than visual cues (Paton et al., 2012, Izawa et al., 2012). Among individuals with ASD, decreased sensitivity to the illusion has been associated with low levels of empathy (Cascio et al., 2012). At the neural level, there is evidence for asynchrony between intrinsic motor and visual brain networks in ASD; such neural differences were related to social functioning and have been postulated to underlie the imitation deficits in this population (Nebel et al., 2016). Alterations in the ability to integrate proprioceptive cues with visual inputs could greatly impact imitation, which is crucial for the development of many social functions.

### 6.4 Developmental considerations of impairments in multisensory integration

The real impact of a deficit in multisensory integration likely lies in its cascading effects on the ability to detect and focus on salient social information throughout development. There is converging evidence that infants can detect amodal information − such as space, time, and intensity − at very young ages (Lewkowicz, 2000, Lewkowicz, 2010, Bahrick and Pickens, 1994) and find stimuli particularly salient when amodal information is synchronously available to multiple senses [known as inter-sensory redundancy] (Bahrick and Lickliter, 2000, Bahrick et al., 2004). It has been proposed (Bahrick, 2010, Bahrick and Todd, 2012) that social stimuli − such as speech, faces, voices − inherently provide large amounts of inter-sensory redundancy, resulting in preferences for social over nonsocial stimuli in infants (Farah et al., 1998); there is evidence that these preferences are altered in ASD (Swettenham et al., 1998). Thus, the ability to detect amodal information is a prerequisite for sensory integration; alterations in early integration abilities could limit the salience of social stimuli and cascade into social deficits across development.

Interoception, which involves processing of self in terms of bodily functions and their sensory integration, might produce a more basic, lower-order processing of self in terms of bodily functions, visceral sensations (such as temperature, stretch and pain from the gut, light nondiscriminatory touch, itch tickle, and hunger; Quattrocki and Friston, 2014) and their sensory integration in constituting self-relatedness and identity (Zaytseva et al., 2014). Poor recruitment of these regions in individuals with ASD may underlie their failure to adopt the bodily-anchored psychological and communicative stance of another person (Hobson and Meyer, 2005). Thus, this physical self might be a precursor for developing a more abstract sense of self, and a lack of ability to integrate in ASD populations might be an obstacle in forming the sense of self needed for mastering theory-of-mind (ToM) skills.

## 7 Theoretical models integrating sensory and social features of ASD

### 7.1 Temporal binding hypothesis

The temporal binding hypothesis (Brock et al., 2002) has previously been used to explain altered sensory functioning in ASD. It is based on the premise that sensory stimuli that occur in close temporal proximity are more likely to be integrated and perceived as emanating from the same source; thus, timing information is crucial to binding and integrating associated stimuli (Shams et al., 2000, McGurk and Macdonald, 1976, Stevenson et al., 2012). There is converging evidence that the “temporal binding window” is extended in individuals with ASD, which may give rise to alterations in sensory processing (see Wallace and Stevenson, 2014 for a review; Foss-Feig et al., 2010, Kwakye et al., 2011, Stevenson et al., 2014b). A longer temporal binding window could create a “fuzzier”, unpredictable sensory environment (Wallace and Stevenson, 2014), as unrelated stimuli become bound together. Throughout development, important social cues may fail to become integrated or salient. For example, the concurrent lip movement and voice of a parent calling a child’s name may not become salient over the other co-occurring stimuli in the environment. This would affect social responses and potentially lead to a preference for restrictive, repetitive behaviors as a refuge from the unpredictable social environment (Johnson et al., 2015). An altered temporal binding window could also impact social learning, such as those involving rewards. Reward learning requires critically timed and predictable co-occurrence of stimuli, and an abnormal ability to bind neutral cues with rewards and punishments would be detrimental to social development. There is evidence of altered stimulus-reward associations in ASD (Dawson et al., 2001, Dawson et al., 2002, Zalla et al., 2009, Kohls et al., 2011). Thus, an extended temporal binding window could negatively impact social behavior in ASD through altered binding of social cues.

### 7.2 Intense world theory

The Intense World Theory offers another mechanism for how the sensory and social features of ASD may be related (Markram et al., 2007, Markram and Markram, 2010). This neurobiologically-informed theory proposes that there is excessive functioning of neural circuits, such that the neural circuits are hyper-reactive, hyper-plastic, and generally up-regulated. This creates an intense world, a fragmented world (with focus on individual components of the environment), and an aversive world. Low-level sensory perception is enhanced (intense world), coupled with deficits in sensory integration (fragmented world). Such perceptual up-regulation results in an avoidance of highly emotional and unpredictable cues, such as eyes, faces, and social interactions. This results in eye gaze aversion, social withdrawal, limited communication, and a focus on stable, predictable cues instead (Markram and Markram, 2010). Throughout development, this could lead to an over-specialization for perceiving primary sensory cues at the expense of the ability to navigate in a socially complex world (Markram and Markram, 2010). In this way, the Intense World Theory explains both the unique sensory and social features of ASD and offers a mechanism for how an up-regulation in primary sensory perception results in social avoidance and withdrawal.

### 7.3 Atypical hierarchical information processing

Atypical hierarchical information processing may hinder sensory and social functioning in individuals with ASD. To efficiently perceive and operate in a dynamic world, humans use both incoming sensory information (bottom-up processes) and inference from prior experience and context (top-down processes) (e.g., Knill and Pouget, 2004). Research has suggested that under-utilization of top-down processes such as context or experience (e.g., Pellicano and Burr, 2012) or an over-reliance on bottom-up sensory perception (e.g., Brock, 2012a, Mottron and Burack, 2001, Mottron et al., 2006) characterizes perception in ASD. Predictive coding, hypo-priors, Weak Central Coherence, and Enhanced Perceptual Functioning accounts characterize perception in ASD as under-utilizing top-down processes (context, prior knowledge, or global coherence) or over-functioning of bottom-up sensory processes (Pellicano and Burr, 2012, Van Boxtel and Lu, 2013, Van de Cruys et al., 2012, Lawson et al., 2014, Frith, 1989, Happe and Frith, 2006, Mottron and Burack, 2001, Mottron et al., 2006). These models all predict the superior perception seen at times in ASD, such as reduced susceptibility to illusions, and superior visual search and pitch perception (Happe, 1996, Bonnel et al., 2003, Jolliffe and Baron-Cohen, 1997, Pellicano et al., 2006, Muth et al., 2014), but inefficient perception of ambiguous or complex sensory information. At the neural level, this profile may reflect hyper-activation of primary sensory cortices, decreased prefrontal activity, and reduced neural habituation during sensory processing (Ring et al., 1999, Lee et al., 2007, Kana et al., 2013, Guiraud et al., 2011, Green et al., 2015). This information processing profile may hamper social functioning, as the interpersonal world demands strong central coherence, integration of context, and utilization of prior knowledge. This is supported by evidence that local processing biases and enhanced perceptual abilities negatively predict social skills (Meaux et al., 2011, Russell-Smith et al., 2012). Predictive coding may also underlie mentalizing (Palmer et al., 2015), as individuals with ASD are impaired in using social information to predict others’ actions (von der Luhe et al., 2016). Thus, over-functioning of bottom-up sensory processing coupled with under-utilizing top-down perception in ASD could explain both enhanced sensory processing and inefficient social functioning in this population.

## 8 Neurobiology of the sensory-social axis in ASD

The neurobiological underpinnings of sensory abnormalities in the context of social cognition in autism have been less addressed in the literature. However, many of the atypical functional and anatomical circuits that underlie sensory processing are also implicated in social processing impairments in autism. While several primary sensory and association cortex areas may be involved in sensory processing and integration of that information, we will focus on a few important areas and address their role in sensory and social processing in individuals with ASD. These regions are the: 1) Thalamus, Insula, and Cingulate Cortex; 2) Superior Temporal Sulcus; and 3) Cerebellum.

### 8.1 Thalamus, insula, and cingulate cortex

The thalamus and basal ganglia form circuits throughout the brain that are connected to cognitive, motor, and emotional functioning (Schuetze et al., 2016). The thalamus is a relay center in subserving both sensory and motor mechanisms, and awareness (Smythies, 1997), attention (Büchel et al., 1998) and other neurocognitive processes such as memory and language (Engelborghs et al., 1998, Johnson and Ojemann, 2000). All sensory input with the exception of olfaction passes through the thalamus before reaching their associated primary cortical areas. The thalamus is also believed to have a complex feed-forward and feedback connectivity with cortical and subcortical areas (Sherman, 2007). Neuroimaging studies of the thalamus have found atypical functional and anatomical connectivity (Horwitz et al., 1988, Nair et al., 2013), decreased thalamic volume (Tsatsanis et al., 2003, Tamura et al., 2010), lower levels of *N*-acetylasparate, phosphocreatine, creatin, and choline-containing metabolites (Hardan et al., 2008, Haznedar et al., 2006), and reduced neuronal integrity (Friedman et al., 2003) in ASD. It should be noted that some of these findings, such as lower levels of metabolites, were also associated with higher levels of sensory abnormalities (Hardan et al., 2008). Abnormal connections from thalamus or lesions to this area have been associated with major depressive disorder, irritability, and sadness (Hamilton et al., 2014, Gentilini et al., 1987). Thus, the thalamus may be an important structure in the pathobiology of autism, specifically in sensory and social differences.

The anterior cingulate cortex (ACC) and insula receive input from the thalamus, and are thought to be major nodes of the limbic system which contributes to emotion processing (Hadland et al., 2003), learning (Bush et al., 2000, Devinsky et al., 1995), and memory (Frankland et al., 2004, Sutherland et al., 1988). These areas are also involved in interoceptive awareness (Craig, 2003). Neuroanatomical alterations (Ebisch et al., 2011, Uddin and Menon, 2009, Doyle-Thomas et al., 2013b, Haznedar et al., 1997, Henderson et al., 2006) of these areas in ASD may result in poor ability to integrate the physical self into a self-identity, possibly creating the stereotypical deficit in ToM in autism. This metabolic activity could also play a part in the regulation of affective reactions and forming associations between sensory stimuli and their emotional values (Strata et al., 2011). Relationships between the ACC and insula activation and social interaction in ASD have also been reported (Schmitz et al., 2008, Doyle-Thomas et al., 2013b). These findings suggest the role of ACC and insula in both the sensory and social impairments observed in ASD.

### 8.2 Superior temporal cortex

The superior temporal cortex (STC), including the superior temporal sulcus and gyrus, is considered a hub of the “social brain” network (Pelphrey and Carter, 2008), with important roles in emotion recognition (Narumoto et al., 2001), understanding intention (Pelphrey et al., 2004a, Castelli et al., 2002, Kana et al., 2009, Kana et al., 2015), biological motion perception (Allison et al., 2000) and gaze detection (Pelphrey et al., 2004b, Mosconi et al., 2005, Saitovitch et al., 2016), amongst other social-cognitive skills. The STC may be abnormally developed in ASD, with reductions in overall volume, decreased activity during social tasks, and decreased connectivity with other regions (Boddaert et al., 2004, Patriquin et al., 2016, Venkataraman et al., 2015, Shih et al., 2011). Further, the functional connectivity difference in STC has been found to predict emotion recognition and other social difficulties in ASD (Chien et al., 2015, Alaerts et al., 2014). STC dysfunction during social interactions is seen across sensory modalities and in many of the sensory-linked social impairments discussed above; for example, STC hypo-activation has been documented in ASD during biological motion detection (Pelphrey et al., 2007), speech perception (Redcay, 2008), processing affective touch (Kaiser et al., 2016), and integrating auditory and visual speech information (Stevenson et al., 2011, Loveland et al., 2008).

The STC also underlies many non-social sensory and perceptual functions, including conscious perception of visual motion, listening to both meaningful and non-meaningful sounds, and multisensory integration (Becker et al., 2013, Lewis et al., 2004, Lapenta et al., 2012). Reduced STC activity or connectivity has also been documented in non-social sensory processing in ASD, including listening to tones and perceptual integration (Samson et al., 2011, Edgar et al., 2014, Peiker et al., 2015). In addition to the role of STC in sensory and social processes, it has been hypothesized that the many connections of the STC with primary sensory cortices, multimodal associative systems, and the limbic system, may explain its role in a diverse range of functions (Boddaert and Zilbovicius, 2002). It has also been proposed that the STC may play a common role across its many associated functions such that it processes and integrates various modes of incoming sensory information in order to assign meaning to one’s world (Redcay, 2008, Jou et al., 2010). Thus, the critical role of the STC in perceptual and social functioning in ASD makes it an important candidate for understanding the complex symptomatology of autism.

### 8.3 Cerebellum

The cerebellum, consistently implicated in ASD neuropathology (Fatemi et al., 2012), perhaps explains the co-occurrence of sensory and social deficits in ASD. An overarching description is that the cerebellum is a “co-processor”, modulating diverse regions and functions of the brain through feedback loops (Wolpert et al., 1998, D'Angelo and Casali, 2012); in this way, it can impact both sensory and social functions. The cerebellum has been proposed to enhance and depress sensory stimuli and could contribute to abnormal sensory perception in ASD (Kern, 2002). The cerebellum is functionally connected with different cortical nodes, allowing it to modulate diverse functions − ranging from sensory to social − through feedback loops (Wolpert et al., 1998, D'Angelo and Casali, 2012). Specifically, the interaction of the cerebellum with higher-order intrinsic connectivity networks (Habas et al., 2009, Krienen and Buckner, 2009) could similarly modulate executive function, mentalizing, and salience detection − functions critical for social cognition and social interaction (Menon, 2011). In fact, the cerebellum is involved in language, emotion, and social cognition specifically (Stoodley and Schmahmann, 2009, Van Overwalle et al., 2014). The cerebellum also forms feedback loops with multisensory regions such as the superior colliculus (SC). Abnormal cerebellar-SC connectivity could lead to altered eye contact and orientation to stimuli, functions controlled by the SC (Kern, 2002, Quaia et al., 2012). Thus, extensive connections of the cerebellum with brain regions and networks primarily associated with sensory and social processing could allow it to simultaneously modulate behaviors within each domain.

The cerebellum also has an important role in timing, prediction, and learning (Ivry and Keele, 1989, Baumann et al., 2015). Alterations in timing could impair integration of temporally synchronous stimuli (Wallace and Stevenson, 2014), which would have profound effects on sensory perception and ultimately social responses (D'Angelo and Casali, 2012). Such abnormalities in timing are apparent in the extended temporal binding window in ASD (Foss-Feig et al., 2010). The role of the cerebellum in prediction and learning also critically impacts sensory and social processes. The cerebellum operates in a feed-forward manner (Wolpert et al., 1998, Miall et al., 1993), making predictions about the environment and learning from error signals (Marr, 1969) to optimize behavior. In this way, the cerebellum utilizes internal models to dynamically coordinate behavior during social interactions in a way similar to motor control (Wolpert et al., 2003, Ito, 2008, D'Angelo and Casali, 2012). Inability of the cerebellum to either integrate social cues, or to form internal models based on these cues, would have ultimate effects on social behavior.

## 9 Conclusion

Sensory abnormalities are one of the earliest emerging markers of infants later diagnosed with ASD, with differences noted as early as 6-months of age (Clifford et al., 2013). The sensory characteristics seen across ASD are heterogeneous, with many features ranging from intact, enhanced, or impaired from one sample to the next. Despite these inconsistencies, dysregulated sensory processing can be considered universal in ASD. This paper consolidated the evidence emerging from behavior, neuroscience, and other modalities of research on sensory to social processing in ASD in order to establish their inter-relationship and impact on ASD symptomatology. Abnormal sensory sensitivity in ASD has significant clinical and social implications. Clinically, atypical sensory processing in ASD can interfere with obtaining an accurate assessment of skills, progression of therapy, and treatment outcomes. Oversensitivity to perceptual level sensory features can come at the expense of inability to filter out extraneous information and selectively attend to instruction in the therapeutic environment. Conversely, hyposensitivity to sensory stimuli in the environment can result in delayed visual and auditory processing, lack of appropriate response, and poor multisensory integration. Socially, this profile of sensory sensitivity can impact selective attention to social stimuli, decoding intentions, social reciprocity, and adherence to social norms of behavior. In general, sensory issues, which may differ across individuals on the autism spectrum, take an important role in social and communicative difficulties in ASD, and hence future research needs to consider it seriously while designing treatment plans for children with ASD.

There are several cognitive and neurobiological mechanisms through which sensory processing abnormalities might either cause or exacerbate many of the social impairments seen in autism. Functional and anatomical differences seen in the thalamus of individuals with ASD may be central to this considering the role of thalamus as a relay station for most senses. Furthermore, abnormalities found in regions such as the ACC, insula and STC in ASD also point to a network of regions at the intersection of sensory processing and social cognition. The cerebellum also plays a complex role in sensory feedback and integration of social cues within the environment. We find that altered sensory processing and sensory integration in autism affect language, communication, emotion, response to reward, and interpersonal functioning in individuals with ASD. Early intervention is instrumental in altering the developmental progression of ASD (see Reichow, 2012), suggesting the need for early identification of sensory abnormalities as well as other predictors of the disorder. It is also important to continue building models of ASD that incorporate both the social and non-social features of the disorder, and also to design individualized interventions which address both social/communicative and sensory processing. In this way, the two core symptom domains of autism are interrelated and require intervention that targets both domains in conjunction.

## Conflict of Interest

None.

## Funding source

This project was funded by the UAB Department of Psychology Faculty Funds and the McNulty-Civitan Scientist Award.

## References

[bib0005] Adolphs R. (2009). The social brain: neural basis of social knowledge. Annu. Rev. Psychol..

[bib0010] Alaerts K., Woolley D.G., Steyaert J., Di Martino A., Swinnen S.P., Wenderoth N. (2014). Underconnectivity of the superior temporal sulcus predicts emotion recognition deficits in autism. Soc. Cogn. Affect. Neurosci..

[bib0015] Allely C.S. (2013). Pain sensitivity and observer perception of pain in individuals with autistic spectrum disorder. Sci. World J..

[bib0020] Allison T., Puce A., McCarthy G. (2000). Social perception from visual cues: role of the STS region. Trends Cogn. Sci..

[bib0025] American Psychiatric Association (2013). Diagnostic and Statistical Manual of Mental Disorders.

[bib0030] Anagnostou E., Soorya L., Brian J., Dupuis A., Mankad D., Smile S., Jacob S. (2014). Intranasal oxytocin in the treatment of autism spectrum disorders: a review of literature and early safety and efficacy data in youth. Brain Res..

[bib0035] Annaz D., Campbell R., Coleman M., Milne E., Swettenham J. (2012). Young children with autism spectrum disorder do not preferentially attend to biological motion. J. Autism Dev. Disord..

[bib0040] Ashwin C., Ricciardelli P., Baron-Cohen S. (2009). Positive and negative gaze perception in autism spectrum conditions. Soc. Neurosci..

[bib0045] Atkinson A.P. (2009). Impaired recognition of emotions from body movements is associated with elevated motion coherence thresholds in autism spectrum disorders. Neuropsychologia.

[bib0050] Bachevalier J., Loveland K.A. (2006). The orbitofrontal–amygdala circuit and self-regulation of social–emotional behavior in autism. Neurosc. Biobehav. Rev..

[bib0055] Bahrick L.E. (2010).

[bib0060] Bahrick L.E., Lickliter R. (2000). Intersensory redundancy guides attentional selectivity and perceptual learning in infancy. Dev. Psychol..

[bib0065] Bahrick L.E., Pickens J.N. (1994). Amodal relations: the basis for intermodal perception and learning. In The Development of Intersensory Perception: Comparative Perspectives.

[bib0070] Bahrick L.E., Lickliter R., Flom R. (2004). Intersensory redundancy guides the development of selective attention, perception, and cognition in infancy. Current Directions in Psychological Science.

[bib0075] Bahrick L., Todd J. (2012). Multisensory processing in autism spectrum disorders: Intersensory processing disturbance as a basis for atypical development. The New Handbook of Multisensory Processes.

[bib0080] Baranek G. (1999). Autism during infancy: a retrospective video analysis of sensory-motor and social behaviors at 9–12 months of age. J. Autism Dev. Disord..

[bib0085] Baranek G.T., Watson L.R., Boyd B.A., Poe M.D., David F.J., McGuire L. (2013). Hyporesponsiveness to social and nonsocial sensory stimuli in children with autism, children with developmental delays, and typically developing children. Dev Psychopathology.

[bib0090] Barnett K. (1972). A theoretical construct of the concepts of touch as they relate to nursing. Nurs. Res..

[bib0095] Baron-Cohen S. (1990). Autism: a specific cognitive disorder of mind-Blindness. Int. Rev. Psychiatry.

[bib0100] Baron-Cohen S. (1991). Do people with autism understand what causes emotion?. Child Dev..

[bib0105] Baron-Cohen S. (1997). Mindblindness: An Essay on Autism and Theory of Mind.

[bib0110] Baron-Cohen S., Leslie A.M., Frith U. (1985). Does the autistic child have a theory of mind?. Cognition.

[bib0115] Baum S.H., Stevenson R.A., Wallace M.T. (2015). Behavioral, perceptual, and neural alterations in sensory and multisensory function in autism spectrum disorder. Prog. Neurobiol..

[bib0120] Baumann O., Borra R.J., Bower J.M., Cullen K.E., Habas C., Ivry R.B. (2015). Consensus paper: the role of the cerebellum in perceptual processes. Cerebellum.

[bib0125] Bebko J.M., Schroeder J.H., Weiss J.A. (2014). The McGurk effect in children with autism and Asperger syndrome. Autism Research.

[bib0130] Becker H.G., Haarmeier T., Tatagiba M., Gharabaghi A. (2013). Electrical stimulation of the human homolog of the medial superior temporal area induces visual motion blindness. J. Neurosci..

[bib0135] Bedford R., Elsabbagh M., Gliga T., Pickles A., Senju A., Charman T., Johnson M.H. (2012). Precursors to social and communication difficulties in infants at-risk for autism: gaze following and attentional engagement. J. Autism Dev. Disord..

[bib0140] Behrmann M., Thomas C., Humphreys K. (2006). Seeing it differently: visual processing in autism. Trends Cogn. Sci..

[bib0145] Belin P., Fecteau S., Bedard C. (2004). Thinking the voice: neural correlates of voice perception. Trends Cogn. Sci..

[bib0150] Bennetto L., Kuschner E., Hyman S. (2007). Olfaction and taste processing in autism. Biol. Psychiatry.

[bib0155] Bennetto L., Zampella C., Kuschner E., Bender R., Hyman S. (2012). Food preferences in autism spectrum disorders and their relationship to sensory and behavioral symptoms. International Meeting for Autism Research.

[bib0160] Ben-Sasson A., Cermak S.A., Orsmond G.I., Tager-Flusberg H. (2007). Extreme sensory modulation behaviors in toddlers with autism spectrum disorders. Am. J. Occup. Ther..

[bib0165] Bertone A., Mottron L., Jelenic P., Faubert J. (2003). Motion perception in autism: a complex issue. J. Cogn. Neurosci..

[bib0170] Björnsdotter M., Olausson H. (2011). Vicarious responses to social touch in posterior insular cortex are tuned to pleasant caressing speeds. J. Neurosci..

[bib0175] Björnsdotter M., Gordon I., Pelphrey K., Olausson H., Kaiser M. (2014). Development of brain mechanisms for processing affective touch. Frontiers in behavioral neuroscience.

[bib0180] Björnsdotter M., Löken L., Olausson H., Vallbo Å., Wessberg J. (2009). Somatotopic organization of gentle touch processing in the posterior insular cortex. J. Neurosci..

[bib0185] Blake R., Turner L.M., Smoski M.J., Pozdol S.L., Stone W.L. (2003). Visual recognition of biological motion is impaired in children with autism. Psychol. Sci..

[bib0190] Blakemore S.J., Tavassoli T., Calò S., Thomas R.M., Catmur C., Frith U., Haggard P. (2006). Tactile sensitivity in Asperger syndrome. Brain Cogn..

[bib0195] Boddaert N., Chabane N., Belin P., Bourgeois M., Royer V., Barthelemy C., Zilbovicius M. (2004). Perception of complex sounds in autism: abnormal auditory cortical processing in children. Am. J. Psychiatry.

[bib0200] Boddaert N., Zilbovicius M. (2002). Functional neuroimaging and childhood autism. Pediatr. Radiol..

[bib0205] Bölte S., Poustka F. (2003). The recognition of facial affect in autistic and schizophrenic subjects and their first-degree relatives. Psychol. Med..

[bib0210] Bolte S., Holtmann M., Poustka F., Scheurich A., Schmidt L. (2007). Gestalt perception and local-global processing in high-functioning autism. J. Autism Dev. Disord..

[bib0215] Bonnel A., McAdams S., Smith B., Berthiaume C., Bertone A., Ciocca V., Burack J.A., Mottron L. (2010). Enhanced pure-tone pitch discrimination among persons with autism but not Asperger syndrome. Neuropsychologia.

[bib0220] Bonnel A., Mottron L., Peretz I., Trudel M., Gallun E., Bonnel A.-M. (2003). Enhanced pitch sensitivity in individuals with autism: a signal detection analysis. J. Cogn. Neurosci..

[bib0225] Bouvet L., Donnadieu S., Valdois S., Caron C., Dawson M., Mottron L. (2014). Veridical mapping in savant abilities, absolute pitch, and synesthesia: an autism case study. Front. Psychol..

[bib0230] Brandwein A.B., Foxe J.J., Butler J.S., Frey H.P., Bates J.C., Shulman L.H., Molholm S. (2015). Neurophysiological indices of atypical auditory processing and multisensory integration are associated with symptom severity in autism. J. Autism Dev. Disord..

[bib0235] Brandwein A.B., Foxe J.J., Butler J.S., Russo N.N., Altschuler T.S., Gomes H., Molholm S. (2013). The development of multisensory integration in high-functioning autism: high-density electrical mapping and psychophysical measures reveal impairments in the processing of audiovisual inputs. Cereb. Cortex.

[bib0240] Brang D., Ramachandran V. (2010). Olfactory bulb dysgenesis, mirror neuron system dysfunction, and autonomic dysregulation as the neural basis for autism. Med. Hypotheses.

[bib0245] Brauer J., Xiao Y., Poulain T., Friederici A., Schirmer A. (2016). Frequency of maternal touch predicts resting activity and connectivity of the developing social brain. Cereb. Cortex.

[bib0250] Brock J. (2012). Alternative Bayesian accounts of autistic perception: comment on Pellicano and Burr. Trends Cogn. Sci..

[bib0255] Brock J., Brown C.C., Boucher J., Rippon G. (2002). The temporal binding deficit hypothesis of autism. Dev. Psychopathol..

[bib0260] Brock M.E., Freuler A., Baranek G.T., Watson L.R., Poe M.D., Sabatino A. (2012). Temperament and sensory features of children with autism. J Autism Dev Disord.

[bib0265] Brothers L. (2002). The social brain: a project for integrating primate behavior and neurophysiology in a new domain. Found. Docial Neurosci..

[bib0270] Brown N.B., Dunn W. (2010). Relationship between context and sensory processing in children with autism. Am. J. Occup. Ther..

[bib0275] Büchel C., Josephs O., Rees G., Turner R., Frith C.D., Friston K.J. (1998). The functional anatomy of attention to visual motion. A functional MRI study. Brain.

[bib0280] Bush G., Luu P., Posner M.I. (2000). Cognitive and emotional influences in anterior cingulate cortex. Trends Cogn. Sci..

[bib0285] Butcher C., Goldin-Meadow S., McNeill D. (2000). Gesture and the transition from one- to two-word speech: when hand and mouth come together. Lang. Gesture.

[bib0290] Butts J. (2001). Outcomes of comfort touch in institutionalized elderly female residents Geriatric. Nursing (Lond.).

[bib0295] Campbell D.B., Datta D., Jones S.T., Lee E.B., Sutcliffe J.S., Hammock E.A., Levitt P. (2011). Association of oxytocin receptor (OXTR) gene variants with multiple phenotype domains of autism spectrum disorder. J. Neurodevel. Disord..

[bib0300] Capps L., Sigman M., Mundy P. (1994). Attachment security in children with autism. Dev. Psychopathol..

[bib0305] Cascio C.J., Foss-Feig J.H., Burnette C.P., Heacock J.L., Cosby A.A. (2012). The rubber hand illusion in children with autism spectrum disorders: delayed influence of combined tactile and visual input on proprioception. Autism.

[bib0310] Cascio C., McGlone F., Folger S., Tannan V., Baranek G., Pelphrey K.A., Essick G. (2008). Tactile perception in adults with autism: a multidimensional psychophysical study. J. AutismDev. Disord..

[bib0315] Castelli F., Frith C., Happe F., Frith U. (2002). Autism, Asperger syndrome and brain mechanisms for the attribution of mental states to animated shapes. Brain.

[bib0320] Ceponiene R., Lepistö T., Shestakova A., Vanhala R., Alku P., Näätänen R., Yaguchi K. (2003). Speech-sound-selective auditory impairment in children with autism: they can perceive but do not attend. Proc. Natl. Acad. Sci. U. S. A..

[bib0325] Charbonneau G., Bertone A., Lepore F., Nassim M., Lassonde M., Mottron L., Collignon O. (2013). Multilevel alterations in the processing of audio-visual emotion expressions in autism spectrum disorders. Neuropsychologia.

[bib0330] Charman T., Swettenham J., Baron-Cohen S., Cox A., Baird G., Drew A. (1997). Infants with autism: an investigation of empathy, pretend play, joint attention, and imitation. Dev. Psychol..

[bib0335] Charrier A., Tardif C., Gepner B. (2016). Slowing down the flow of facial information enhances facial scanning in children with autism spectrum disorders: a pilot eye tracking study. L'Encephale.

[bib0340] Chien H.Y., Lin H.Y., Lai M.C., Gau S.S., Tseng W.Y. (2015). Hyperconnectivity of the right posterior temporo-parietal junction predicts social difficulties in boys with autism spectrum disorder. Autism Res..

[bib0345] Christensen D.L., Baio J., Braun K.V.N., Bilder D., Charles J., Constantino J.N. (2016). Prevalence and characteristics of autism spectrum disorder among children aged 8 years-Autism and developmental disabilities monitoring network, 11 sites, United States, 2012. MMWR Surveill. Summ..

[bib0350] Clifford S.M., Hudry K., Elsabbagh M., Charman T., Johnson M.H., BASIS Team (2013). Temperament in the first 2 years of life in infants at high-risk for autism spectrum disorders. J. Autism Dev. Disord..

[bib0355] Collignon O., Charbonneau G., Peters F., Nassim M., Lassonde M., Lepore F., Bertone A. (2013). Reduced multisensory facilitation in persons with autism. Cortex.

[bib0360] Craig A.D. (2003). Interoception: the sense of the physiological condition of the body. Curr. Opin. Neurobiol..

[bib0365] Crane L., Goddard L., Pring L. (2009). Sensory processing in adults with autism spectrum disorders. Autism.

[bib0370] Csibra G., Hernik M., Mascaro O., Tatone D., Lengyel M. (2016). Statistical treatment of looking-time data. Dev. Psychol..

[bib0375] Curtin S., Vouloumanos A. (2013). Speech preference is associated with autistic-like behavior in 18-months-olds at risk for Autism Spectrum Disorder. J. Autism Dev. Disord..

[bib0380] D’Angelo E., Casali S. (2012). Seeking a unified framework for cerebellar function and dysfunction: from circuit operations to cognition. Front. Neural Circuits.

[bib0385] Dakin S., Frith U. (2005). Vagaries of visual perception in autism. Neuron.

[bib0390] Dawson G., Meltzoff A.N., Osterling J., Rinaldi J., Brown E. (1998). Children with autism fail to orient to naturally occuring social stimuli. J. Autism Dev. Disord..

[bib0395] Dawson G., Munson J., Estes A., Osterling J., McPartland J., Toth K. (2002). Neurocognitive function and joint attention ability in young children with autism spectrum disorder versus developmental delay. Child Dev..

[bib0400] Dawson G., Osterling J., Rinaldi J., Carver L., McPartland J. (2001). Brief report: recognition memory and stimulus-Reward associations: indirect support for the role of ventromedial prefrontal dysfunction in autism. J. Autism Dev. Disord..

[bib0405] Dawson G., Webb S.J., McPartland J. (2005). Understanding the nature of face processing impairment in autism: insights from behavioral and electrophysiological studies. Dev. Neuropsychol..

[bib0410] de Gelder B., Vroomen J. (2000). The perception of emotions by ear and by eye. Cognit. Emotion.

[bib0415] de Gelder B., Vroomen J., van der Heide L. (1991). Face recognition and lip-reading in autism. Eur. J. Cognit. Psychol..

[bib0420] de Marchena A., Eigsti I.-M. (2010). Conversational gestures in autism spectrum disorders: asynchrony but not decreased frequency. Autism Res..

[bib0425] Demopoulos C., Hopkins J., Kopald B.E., Paulson K., Doyle L., Andrews E. (2015). Neuropsychology deficits in auditory processing contribute to impairments in vocal affect recognition in autism spectrum disorders: a MEG study deficits in auditory processing contribute to impairments in vocal affect recognition in autism. Spect. Disord..

[bib0430] Devinsky O., Morrell M.J., Vogt B.A. (1995). Contributions of anterior cingulate cortex to behavior. Brain.

[bib0435] Dodd B. (1979). Lip reading in infants: attention to speech presented in- and out-of-synchrony. Cognit. Psychol..

[bib0440] Donohue S.E., Darling E.F., Mitroff S.R. (2012). Links between multisensory processing and autism. Exp. Brain Res..

[bib0445] Dowd A.M., McGinley J.L., Taffe J.R., Rinehart N.J. (2012). Do planning and visual integration difficulties underpin motor dysfunction in autism? A kinematic study of young children with autism. J. Autism Dev. Disord..

[bib0450] Doyle-Thomas K.R., Goldberg J., Szatmari P., Hall G.B.C. (2013). Neurofunctional underpinnings of audiovisual emotion processing in teens with autism spectrum disorders. Front. Psychiatry.

[bib0455] Doyle-Thomas K.A., Kushki A., Duerden E.G., Taylor M.J., Lerch J.P., Soorya L.V., Wang A.T., Fan J., Anagnostou E. (2013). The effect of diagnosis, age, and symptom severity on cortical surface area in the cingulate cortex and insula in autism spectrum disorders. J. Child Neurol..

[bib0460] Dudova I., Vodicka J., Havlovicova M., Sedlacek Z., Urbanek T., Hrdlicka M. (2011). Odor detection threshold, but not odor identification, is impaired in children with autism. Eur. Child Adol. Psychiatry.

[bib0465] Dunbar R. (2010). The social role of touch in humans and primates: behavioural function and neurobiological mechanisms. Biobehav. Rev..

[bib0470] Dunn M.A., Gomes H., Gravel J. (2008). Mismatch negativity in children with autism and typical development. J. Autism Dev. Disord..

[bib0475] Ebisch S.J., Gallese V., Willems R.M., Mantini D., Groen W.B., Romani G.L., Buitelaar J.K., Bekkering H. (2011). Altered intrinsic functional connectivity of anterior and posterior insula regions in high-functioning participants with autism spectrum disorder. Hum. Brain Mapp..

[bib0480] Ebstein R.P., Israel S., Lerer E., Uzefovsky F., Shalev I., Gritsenko I. (2009). Arginine vasopressin and oxytocin modulate human social behavior. Ann. N. Y. Acad. Sci..

[bib0485] Edgar J.C., Lanza M.R., Daina A.B., Monroe J.F., Khan S.Y., Blaskey L. (2014). Missing and delayed auditory responses in young and older children with autism spectrum disorders. Front. Hum. Neurosci..

[bib0490] Edwards L.A. (2014). A meta-analysis of imitation abilities in individuals with autism spectrum disorders. Autism Res..

[bib0495] Eigsti I.-M., Fein D.A. (2013). More is less: pitch discrimination and language delays in children with optimal outcomes from autism. Autism Res..

[bib0500] Eigsti I.-M., Schuh J., Mencl E., Schultz R.T., Paul R. (2012). The neural underpinnings of prosody in autism. Child Neuropsychol..

[bib0505] Elsabbagh M., Fernandes J., Jane Webb S., Dawson G., Charman T., Johnson M.H. (2013). Disengagement of visual attention in infancy is associated with emerging autism in toddlerhood. Biol. Psychiatry.

[bib0510] Elsabbagh M., Gliga T., Pickles A., Hudry K., Charman T., Johnson M.H. (2013). The development of face orienting mechanisms in infants at-risk for autism. Behav. Brain Res..

[bib0515] Engelborghs S., Marien P., Martin J.J., De Deyn P.P. (1998). Functional anatomy, vascularisation and pathology of the human thalamus. Acta Neurol. Belg..

[bib0520] Ewing L., Pellicano E., Rhodes G. (2013). Reevaluating the selectivity of face-processing difficulties in children and adolescents with autism. J. Exp. Child Psychol..

[bib0525] Fan Y.T., Cheng Y. (2014). Atypical mismatch negativity in response to emotional voices in people with autism spectrum conditions. PLoS One.

[bib0530] Farah M.J., Wilson K.D., Drain M., Tanaka J.N. (1998). What is special about face perception?. Psychol. Rev..

[bib0535] Fatemi S.H., Aldinger K.A., Ashwood P., Bauman M.L., Blaha C.D., Blatt G.J. (2012). Consensus paper: pathological role of the cerebellum in Autism. Cerebellum.

[bib0540] Field T.M. (1998). Touch therapy effects on development. International Journal of Behavioral Development.

[bib0545] Forgeot d'Arc B., Delorme R., Zalla T., Lefebvre A., Amsellem F., Moukawane S. (2016). Gaze direction detection in autism spectrum disorder. Autism.

[bib0550] Foss-Feig J.H., Heacock J.L., Cascio C.J. (2012). Tactile responsiveness patterns and their association with core features in autism spectrum disorders. Res. Autism Spect. Disord..

[bib0555] Foss-Feig J.H., Kwakye L.D., Cascio C.J., Burnette C.P., Kadivar H., Stone W.L., Wallace M.T. (2010). An extended multisensory temporal binding window in autism spectrum disorders. Exp. Brain Res..

[bib0560] Foster N.E., V, Ouimet T., Tryfon A., Doyle-Thomas K., Anagnostou E., Hyde K.L. (2016). Effects of age and attention on auditory Global–Local processing in children with autism spectrum disorder. J. Autism Dev. Disord..

[bib0565] Foxe J.J., Schroeder C.E. (2005). The case for feedforward multisensory convergence during early cortical processing. Neuroreport.

[bib0570] Foxe J.J., Molholm S., Del Bene V.A., Frey H.P., Russo N.N., Blanco D., Ross L.A. (2015). Severe multisensory speech integration deficits in high-functioning school-aged children with autism spectrum disorder (ASD) and their resolution during early adolescence. Cerebral Cortex.

[bib0575] Foxton J.M., Stewart M.E., Barnard L., Rodgers J., Young A.H., O'Brien G., Griffiths T.D. (2003). Absence of auditory global interference in autism. Brain.

[bib0580] Frankland P.W., Bontempi B., Talton L.E., Kaczmarek L., Silva A.J. (2004). The involvement of the anterior cingulate cortex in remote contextual fear memory. Science.

[bib0585] Franklin A., Sowden P., Burley R., Notman L., Alder E. (2008). Color perception in children with autism. J. Autism Dev. Disord..

[bib0590] Freitag C.M., Konrad C., Haberlen M., Kleser C., von Gontard A., Reith W., Krick C. (2008). Perception of biological motion in autism spectrum disorders. Neuropsychologia.

[bib0595] Friedman S.D., Shaw D.W., Artru A.A., Richards T.L., Gardner J., Dawson G., Posse S., Dager S.R. (2003). Regional brain chemical alterations in young children with autism spectrum disorder. Neurology.

[bib0600] Frith C.D. (2007). The social brain?. Philos. Trans. R. Soc. Lond. B: Biol. Sci..

[bib0605] Frith U. (1989). Autism: explaining the enigma. Br. J Dev. Psychol..

[bib0610] Frühholz S., Ceravolo L., Grandjean D. (2012). Specific brain networks during explicit and implicit decoding of emotional prosody. Cerebral Cortex (New York, N. Y.: 1991).

[bib0615] Gallagher H.L., Happé F., Brunswick N., Fletcher P.C., Frith U., Frith C.D. (2000). Reading the mind in cartoons and stories: an fMRI study of ‘theory of mind’ in verbal and nonverbal tasks. Neuropsychologia.

[bib0620] Gebauer L., Skewes J., Horlyck L., Vuust P. (2014). Atypical perception of affective prosody in Autism Spectrum Disorder. NeuroImage: Clin..

[bib0625] Gentilini M., De Renzi E.N.N.I.O., Crisi G. (1987). Bilateral paramedian thalamic artery infarcts: report of eight cases Journal of Neurology. Neurosurg. Psychiatry.

[bib0630] Gervais H., Belin P., Boddaert N., Leboyer M., Coez A., Sfaello I., Zilbovicius M. (2004). Abnormal cortical voice processing in autism. Nat. Neurosci..

[bib0635] Ghaziuddin M., Ghaziuddin N., Greden J. (2002). Depression in persons with autism: implications for research and clinical care. J. Autism Dev. Disord..

[bib0640] Gillespie-Lynch K., Khalulyan A., del Rosario M., McCarthy B., Gomez L., Sigman M., Hutman T. (2015). Is early joint attention associated with school-age pragmatic language?. Autism.

[bib0645] Gliga T., Bedford R., Charman T., Johnson M.H. (2015). Enhanced visual search in infancy predicts emerging autism symptoms. Curr. Biol..

[bib0650] Gliga T., Jones E.J., Bedford R., Charman T., Johnson M.H. (2014). From early markers to neuro-developmental mechanisms of autism. Dev. Rev..

[bib0655] Globerson E., Amir N., Kishon-Rabin L., Golan O. (2015). Prosody recognition in adults with high-functioning autism spectrum disorders: from psychoacoustics to cognition. Autism Res..

[bib0660] Goldberg M.C., Lasker A.G., Zee D.S., Garth E., Tien A., Landa R.J. (2002). Deficits in the initiation of eye movements in the absence of a visual target in adolescents with high functioning autism. Neuropsychologia.

[bib0665] Goldstein E.B., Brockmole J.R. (2017). Sensation and Perception.

[bib0670] Gordon I., Voos A., Bennett R., Bolling D., Pelphrey K., Kaiser M. (2013). Brain mechanisms for processing affective touch. Hum. Brain Mapp..

[bib0675] Green L., Fein D., Modahl C., Feinstein C., Waterhouse L., Morris M. (2001). Oxytocin and autistic disorder: alterations in peptide forms. Biol. Psychiatry.

[bib0680] Green S.A., Hernandez L., Tottenham N., Krasileva K., Bookheimer S.Y., Dapretto M. (2015). Neurobiology of sensory overresponsivity in youth with autism spectrum disorders. JAMA Psychiatry.

[bib0685] Greenfield K., Ropar D., Smith A.D., Carey M., Newport R. (2015). Visuo-tactile integration in autism: atypical temporal binding may underlie greater reliance on proprioceptive information. Mol. Autism.

[bib0690] Gregory S.G., Connelly J.J., Towers A.J., Johnson J., Biscocho D., Markunas C.A. (2009). Genomic and epigenetic evidence for oxytocin receptor deficiency in autism. BMC Med..

[bib0695] Guiraud J.A., Kushnerenko E., Tomalski P., Davies K., Ribeiro H., Johnson M.H., BASIS Team (2011). Differential habituation to repeated sounds in infants at high risk for autism. Neuroreport.

[bib0700] Guiraud J.A., Tomalski P., Kushnerenko E., Ribeiro H., Davies K., Charman T., BASIS Team (2012). Atypical audiovisual speech integration in infants at risk for autism. PLoS One.

[bib0705] Gupta M., Gupta A., Watteel G. (1998). Perceived deprivation of social touch in psoriasis is associated with greater psychological morbidity: an index of the stigma experience in dermatologic disorders. Cutis.

[bib0710] Habas C., Kamdar N., Nguyen D., Prater K., Beckmann C.F., Menon V., Greicius M.D. (2009). Distinct Cerebellar Contributions to Intrinsic Connectivity. Networks.

[bib0715] Hadad B., Schwartz S., Maurer D., Lewis T.L. (2015). Motion perception: a review of developmental changes and the role of early visual experience. Front. Integr. Neurosci..

[bib0720] Hadland K.A., Rushworth M.F., Gaffan D., Passingham R.E. (2003). The effect of cingulate lesions on social behavior and emotion. Neuropsychologia.

[bib0725] Haist F., Adamo M., Westerfield M., Courchesne E., Townsend J. (2005). The functional neuroanatomy of spatial attention in autism spectrum disorder. Dev. Neuropsychol..

[bib0730] Hamilton J.P., Chen M.C., Waugh C.E., Joormann J., Gotlib I.H. (2014). Distinctive and common neural underpinnings of major depression, social anxiety, and their comorbidity. Soc. Cogn. Affect. Neurosci..

[bib0735] Hammock E.A., Levitt P. (2006). The discipline of neurobehavioral development: the emerging interface of processes that build circuits and skills. Hum. Dev..

[bib0740] Happe F.G. (1996). Studying weak central coherence at low levels: children with autism do not succumb to visual illusions. A research note. J. Child Psychol. Psychiatry.

[bib0745] Happe F., Frith U. (2006). The weak coherence account: detail-focused cognitive style in autism spectrum disorders. J. Autism Dev. Disord..

[bib0750] Hardan A.Y., Minshew N.J., Melhem N.M., Srihari S., Jo B., Bansal R., Keshavan M.S., Stanley J.A. (2008). An MRI and proton spectroscopy study of the thalamus in children with autism Psychiatry Research. Neuroimaging.

[bib0755] Harms M.B., Martin A., Wallace G.L. (2010). Facial emotion recognition in autism spectrum disorders: a review of behavioral and neuroimaging studies. Neuropsychol. Rev..

[bib0760] Haznedar M.M., Buchsbaum M.S., Hazlett E.A., LiCalzi E.M., Cartwright C., Hollander E. (2006). Volumetric analysis and three-dimensional glucose metabolic mapping of the striatum and thalamus in patients with autism spectrum disorders. Am. J. Psychiatry.

[bib0765] Haznedar M.M., Buchsbaum M.S., Metzger M., Solimando A., Spiegel-Cohen J., Hollander E. (1997). Anterior cingulate gyrus volume and glucose metabolism in autistic disorder. Am. J. Psychiatry.

[bib0770] Heald S.L.M., Nusbaum H.C. (2014). Speech perception as an active cognitive process. Front. Syst. Neurosci..

[bib0775] Heaton P., Williams K., Cummins O., Happé F.G.E. (2007). Beyond perception: musical representation and on-line processing in autism. J. Autism Dev. Disord..

[bib0780] Hellendoorn A., Langstraat I., Wijnroks L., Buitelaar J.K., van Daalen E., Leseman P.P. (2014). The relationship between atypical visual processing and social skills in young children with autism. Res. Dev. Disabil..

[bib0785] Henderson H., Schwartz C., Mundy P., Burnette C., Sutton S., Zahka N., Pradella A. (2006). Response monitoring, the error-related negativity, and differences in social behavior in autism. Brain Cogn..

[bib0790] Hertenstein M. (2002). Touch: its communicative functions in infancy. Hum. Dev..

[bib0795] Hertenstein M., Keltner D., App B., Bulleit B., Jaskolla A. (2006). Touch communicates distinct emotions. Emotion.

[bib0800] Hesling I., Dilharreguy B., Peppé S., Amirault M., Bouvard M., Allard M. (2010). The integration of prosodic speech in high functioning autism: a preliminary fMRI study. PLoS One.

[bib0805] Hilton C.L., Harper J.D., Kueker R.H., Lang A.R., Abbacchi A.M., Todorov A., Lavesser P.D. (2010). Sensory responsiveness as a predictor of social severity in children with high functioning autism spectrum disorders. J. Autism Dev. Disord..

[bib0810] Hobson R.P. (1986). The autistic child's appraisal of expressions of emotion. J. Child Psychol. Psychiatry.

[bib0815] Hobson R.P., Meyer J.A. (2005). Foundations for self and other: a study in autism. Dev. Sci..

[bib0820] Horwitz B., Rumsey J.M., Grady C.L., Rapoport S.I. (1988). The cerebral metabolic landscape in autism: intercorrelations of regional glucose utilization. Arch. Neurol..

[bib0825] Howe F.E., Stagg S.D. (2016). How sensory experiences affect adolescents with an autistic spectrum condition within the classroom. J. Autism Dev. Disord..

[bib0830] Hubbard A.L., Mcnealy K., Scott-Van Zeeland A.A., Callan D.E., Bookheimer S.Y., Dapretto M. (2012). Altered integration of speech and gesture in children with autism spectrum disorders. Brain Behav..

[bib0835] Humphreys K., Hasson U., Avidan G., Minshew N., Behrmann M. (2008). Cortical patterns of category-selective activation for faces, places and objects in adults with autism. Autism Res..

[bib0840] Hyde K.L., Samson F., Evans A.C., Mottron L. (2010). Neuroanatomical differences in brain areas implicated in perceptual and other core features of autism revealed by cortical thickness analysis and voxel-based morphometry. Hum. Brain Mapp..

[bib0845] Iarocci G., McDonald J. (2006). Sensory integration and the perceptual experience of persons with autism. J. Autism Dev. Disord..

[bib0850] Iarocci G., Rombough A., Yager J., Weeks D.J., Chua R. (2010). Visual influences on speech perception in children with autism. Autism: Int. J. Res. Pract..

[bib0855] Ito M. (2008). Control of mental activities by internal models in the cerebellum. Nat. Rev. Neurosci..

[bib0860] Ivry R.B., Keele S.W. (1989). Timing functions of the cerebellum. J. Cogn. Neurosci..

[bib0865] Izawa J., Pekny S.E., Marko M.K., Haswell C.C., Shadmehr R., Mostofsky S.H. (2012). Motor learning relies on integrated sensory inputs in ADHD, but over-selectively on proprioception in autism spectrum conditions. Autism Res..

[bib0870] Jao Keehn R.J., Sanchez S.S., Stewart C.R., Zhao W., Grenesko-Stevens E.L., Keehn B., Müller R.-A. (2016). Impaired downregulation of visual cortex during auditory processing is associated with autism symptomatology in children and adolescents with autism spectrum disorder. Autism Res..

[bib0875] Järvinen-Pasley A., Heaton P. (2007). Evidence for reduced domain-specificity in auditory processing in autism. Dev. Sci..

[bib0880] Järvinen-Pasley A., Peppé S., King-Smith G., Heaton P. (2008). The relationship between form and function level receptive prosodic abilities in autism. J. Autism Dev. Disord..

[bib0885] Johansson G. (1973). Visual perception of biological motion and a model for its analysis. Percept. Psychophys..

[bib0890] Johnson M.D., Ojemann G.A. (2000). The role of the human thalamus in language and memory: evidence from electrophysiological studies. Brain Cogn..

[bib0895] Johnson M.H., Jones E.J.H., Gliga T. (2015). Brain adaptation and alternative developmental trajectories. Dev. Psychopathol..

[bib0900] Jolliffe T., Baron-Cohen S. (1997). Are people with autism and Asperger syndrome faster than normal on the Embedded Figures Test?. J. Child Psychol. Psychiatry.

[bib0905] Jones C.R.G., Happé F., Baird G., Simonoff E., Marsden A.J.S., Tregay J. (2009). Auditory discrimination and auditory sensory behaviors in autism spectrum disorders. Neuropsychologia.

[bib0910] Jones W., Klin A. (2013). Attention to eyes is present but in decline in 2–6-month-old infants later diagnosed with autism. Nature.

[bib0915] Jou R.J., Minshew N.J., Keshavan M.S., Vitale M.P., Hardan A.Y. (2010). Enlarged right superior temporal gyrus in children and adolescents with autism. Brain Res..

[bib0920] Kaiser M.D., Pelphrey K.A. (2012). Disrupted action perception in autism: behavioral evidence, neuroendophenotypes, and diagnostic utility. Dev. Cogn. Neurosci..

[bib0925] Kaiser M.D., Shiffrar M. (2009). The visual perception of motion by observers with autism spectrum disorders: a review and synthesis. Psychon. Bull. Rev..

[bib0930] Kaiser M.D., Yang D.Y., Voos A.C., Bennett R.H., Gordon I., Pretzsch C., Pelphrey K.A. (2016). Brain mechanisms for processing affective (and nonaffective) touch are atypical in autism. Cereb. Cortex.

[bib0935] Källstrand J., Olsson O., Nehlstedt S.F., Sköld M.L., Nielzén S. (2010). Abnormal auditory forward masking pattern in the brainstem response of individuals with Asperger syndrome. Neuropsychiatr. Dis. Treat..

[bib0940] Kana R.K., Keller T.A., Cherkassky V.L., Minshew N.J., Just M.A. (2009). Atypical frontal-posterior synchronization of Theory of Mind regions in autism during mental state attribution. Soc. Neurosci..

[bib0945] Kana R.K., Liu Y., Williams D.L., Keller T.A., Schipul S.E., Minshew N.J., Just M.A. (2013). The local, global, and neural aspects of visuospatial processing in autism spectrum disorders. Neuropsychologia.

[bib0950] Kana R.K., Maximo J.O., Williams D.L., Keller T.A., Schipul S.E., Cherkassky V.L., Just M.A. (2015). Aberrant functioning of the theory-of-mind network in children and adolescents with autism. Mol. Autism.

[bib0955] Kanner L. (1943). Autistic disturbances of affective contact. Nerv. Child.

[bib0960] Kekelis L.S. (1992). Peer interactions in childhood: the impact of visual impairment. Dev. of Soc. Skills Blind Vis. Impaired Students.

[bib0965] Kern J.K. (2002). The possible role of the cerebellum in autism/PDD: Disruption of a multisensory feedback loop. Med. Hypotheses.

[bib0970] Kern J.K., Trivedi M.H., Grannemann B.D., Garver C.R., Johnson D.G., Andrews A.A. (2007). Sensory correlations in autism. Autism.

[bib0975] Kerns C.M., Kendall P.C. (2012). The presentation and classification of anxiety in autism spectrum disorder. Clin. Psychol. Sci. Pract..

[bib0980] Khalfa S., Bruneau N., Rogé B., Georgieff N., Veuillet E., Adrien J.-L., Collet L. (2004). Increased perception of loudness in autism. Hear. Res..

[bib0985] Kientz M.A., Dunn W. (1997). A comparison of the performance of children with and without autism on the Sensory Profile. Am. J. Occup. Ther..

[bib0990] Kirchner J.C., Hatri A., Heekeren H.R., Dziobek I. (2011). Autistic symptomatology, face processing abilities, and eye fixation patterns. J. Autism Dev. Disord..

[bib0995] Klin A. (1991). Young autistic children’s listening preferences in regard to speech: a possible characterization of the symptom of social withdrawal. J. Autism Dev. Disord..

[bib1000] Klin A., Jones W., Schultz R., Volkmar F., Cohen D. (2002). Visual fixation patterns during viewing of naturalistic social situations as predictors of social competence in individuals with autism. Arch. Gen. Psychiatry.

[bib1005] Klin A., Lin D.J., Gorrindo P., Ramsay G., Jones W. (2009). Two-year-olds with autism orient to non-social contingencies rather than biological motion. Nature.

[bib1010] Klin A., Shultz S., Jones W. (2015). Social visual engagement in infants and toddlers with autism: early developmental transitions and a model of pathogenesis. Neurosci. Biobehav. Rev..

[bib1015] Klin A., Sparrow S.S., De Bildt A., Cicchetti D.V., Cohen D.J., Volkmar F.R. (1999). A normed study of face recognition in autism and related disorders. J. Autism Dev. Disord..

[bib1020] Knill D.C., Pouget A. (2004). The Bayesian brain: the role of uncertainty in neural coding and computation. Trends Neurosci..

[bib1025] Kohls G., Peltzer J., Schulte-Rüther M., Kamp-Becker I., Remschmidt H., Herpertz-Dahlmann B., Konrad K. (2011). Atypical brain responses to reward cues in autism as revealed by event-related potentials. J. Autism Dev. Disord..

[bib1030] Koldewyn K., Whitney D., Rivera S.M. (2011). Neural correlates of coherent and biological motion perception in autism. Dev. Sci..

[bib1035] Korpilahti P., Jansson-Verkasalo E., Mattila M.-L., Kuusikko S., Suominen K., Rytky S., Moilanen I. (2007). Processing of affective speech prosody is impaired in Asperger syndrome. J. Autism Dev. Disord..

[bib1040] Krienen F.M., Buckner R.L. (2009). Segregated fronto-cerebellar circuits revealed by intrinsic functional connectivity. Cerebral Cortex.

[bib1045] Kröger A., Bletsch A., Krick C., Siniatchkin M., Jarczok T.A., Freitag C.M., Bender S. (2014). Visual event-related potentials to biological motion stimuli in autism spectrum disorders. Soc. Cogn. Affect. Neurosci..

[bib1050] Kuhl P.K., Coffey-Corina S., Padden D., Dawson G. (2005). Links between social and linguistic processing of speech in preschool children with autism: behavioral and electrophysiological measures. Dev. Sci..

[bib1055] Kumsta R., Hummel E., Chen F.S., Heinrichs M. (2015). Epigenetic regulation of the oxytocin receptor gene: implications for behavioral neuroscience. Soc. Hormones Hum. Behav..

[bib1060] Kwakye L.D., Foss-Feig J.H., Cascio C.J., Stone W.L., Wallace M.T. (2011). Altered auditory and multisensory temporal processing in autism spectrum disorders. Front. Integr. Neurosci..

[bib1065] Kwon S., Kim J., Choe B.H., Ko C., Park S. (2007). Electrophysiologic assessment of central auditory processing by auditory brainstem responses in children with autism spectrum disorders. J. Korean Med. Sci..

[bib1070] Lahera G., Ruiz-Murugarren S., Fernández-Liria A., Saiz-Ruiz J., Buck B.E., Penn D.L. (2016). Relationship between olfactory function and social cognition in euthymic bipolar patients. CNS Spectr..

[bib1075] Lai G., Schneider H.D., Schwarzenberger J.C., Hirsch J. (2011). Speech stimulation during functional MR imaging as a potential indicator of autism. Radiology.

[bib1080] Lane A.E., Dennis S.J., Geraghty M.E. (2011). Brief report: further evidence of sensory subtypes in autism. J. Autism Dev. Disord..

[bib1085] Langland R., Panicucci C. (1982). Effects of touch on communication with elderly confused clients. Nursing (Lond.).

[bib1090] Lapenta O.M., Fregni F., Oberman L.M., Boggio P.S. (2012). Bilateral temporal cortex transcranial direct current stimulation worsens male performance in a multisensory integration task. Neurosci. Lett..

[bib1095] Lawson R.P., Rees G., Friston K.J. (2014). An aberrant precision account of autism. Front. Hum. Neurosci..

[bib1100] Lee K., Eskritt M., Symons L.A., Muir D. (1998). Children's use of triadic eye gaze information for mind reading. Dev. Psychol..

[bib1105] Lee P.S., Foss-Feig J., Henderson J.G., Kenworthy L.E., Gilotty L., Gaillard W.D., Vaidya C.J. (2007). Atypical neural substrates of embedded figures task performance in children with autism spectrum disorder. Neuroimage.

[bib1110] Leekam S. (2016). Social cognitive impairment and autism: what are we trying to explain?. Philos. Trans. R. Soc. Lond. B: Biol. Sci..

[bib1115] Leekam S.R., Nieto C., Libby S.J., Wing L., Gould J. (2007). Describing the sensory abnormalities of children and adults with autism. J. Autism Dev. Disord..

[bib1120] Leekam S., Baron-Cohen S., Perrett D., Milders M., Brown S. (1997). Eye-direction detection: a dissociation between geometric and joint attention skills in autism. Br. J. Devel. Psychol..

[bib1125] Lepistö T., Kuitunen A., Sussman E., Saalasti S., Jansson-Verkasalo E., Nieminen-von Wendt T., Kujala T. (2009). Auditory stream segregation in children with Asperger syndrome. Biol. Psychol..

[bib1130] Lewis J.W., Wightman F.L., Brefczynski J.A., Phinney R.E., Binder J.R., DeYoe E.A. (2004). Human brain regions involved in recognizing environmental sounds. Cereb. Cortex.

[bib1135] Lewkowicz D.J. (2000). The development of intersensory temporal perception: an epigenetic systems/limitations view. Psychol. Bull..

[bib1140] Lewkowicz D.J. (2010). Infant perception of audio-visual speech synchrony. Dev. Psychol..

[bib1145] Lin I.-F., Agus T.R., Suied C., Pressnitzer D., Yamada T., Komine Y. (2016). Fast response to human voices in autism. Sci. Rep..

[bib1150] Linkenauger S.A., Lerner M.D., Ramenzoni V.C., Proffitt D.R. (2012). A perceptual-Motor deficit predicts social and communicative impairments in individuals with autism spectrum disorders. Autism Res..

[bib1155] Liu X., Kawamura Y., Shimada T., Otowa T., Koishi S., Sugiyama T. (2010). Association of the oxytocin receptor (OXTR) gene polymorphisms with autism spectrum disorder (ASD) in the Japanese population. J. Hum. Genetics.

[bib1160] Löken L., Olausson H. (2010). The skin as a social organ. Exp. Brain Res..

[bib1165] Loveland K.A., Steinberg J.L., Pearson D.A., Mansour R., Reddoch S. (2008). Judgments of auditory-visual affective congruence in adolescents with and without autism: a pilot study of a new task using fMRI. Percept. Mot. Skills.

[bib1170] MacLeod A., Summerfield Q. (1987). Quantifying the contribution of vision to speech perception in noise. Br. J. Audiol..

[bib1175] Magnée M.J.C.M., De Gelder B., Van Engeland H., Kemner C. (2007). Facial electromyographic responses to emotional information from faces and voices in individuals with pervasive developmental disorder. J. Child Psychol. Psychiatry.

[bib1180] Magnée M.J.C.M., de Gelder B., van Engeland H., Kemner C. (2011). Multisensory integration and attention in Autism Spectrum disorder: evidence from event-related potentials. PLoS One.

[bib1185] Magnée M.J., de Gelder B., van Engeland H., Kemner C. (2008). Atypical processing of fearful face-voice pairs in Pervasive Developmental Disorder: an ERP study. Clin. Neurophysiol..

[bib1190] Mammen M.A., Moore G.A., Scaramella L.V., Reiss D., Ganiban J.M., Shaw D.S., Leve L., Neiderhiser J.M. (2015). Infant avoidance during a tactile task predicts autism spectrum behaviors in toddlerhood. Infant Mental Health J..

[bib1195] Manning C., Charman T., Pellicano E. (2013). Processing slow and fast motion in children with autism spectrum conditions. Autism Res..

[bib1200] Marco E.J., Hinkley L.B., Hill S.S., Nagarajan S.S. (2011). Sensory processing in autism: a review of neurophysiologic findings. Pediatr. Res..

[bib1205] Markram H., Rinaldi T., Markram K. (2007). The intense world syndrome-an alternative hypothesis for autism. Front. Neurosci..

[bib1210] Markram K., Markram H. (2010). The intense world theory − a unifying theory of the neurobiology of autism. Front. Hum. Neurosci..

[bib1215] Marr D. (1969). A theory of cerebellar cortex. J. Physiol..

[bib1220] Massaro D.W., Egan P.B. (1996). Perceiving affect from the voice and the face. Psychon. Bull. Rev..

[bib1225] Maurer D., Salapatek P. (1976). Developmental changes in the scanning of faces by young infants. Child Dev..

[bib1230] Mayer J.L., Heaton P.F. (2014). Age and sensory processing abnormalities predict declines in encoding and recall of temporally manipulated speech in high-functioning adults with ASD. Autism Res..

[bib1235] McCleery J.P., Allman E., Carver L.J., Dobkins K.R. (2007). Abnormal magnocellular pathway visual processing in infants at risk for autism. Biol. Psychiatry.

[bib1240] McGaha C.G., Farran D.C. (2001). Interactions in an inclusive classroom: the effects of visual status and setting. J. Vis. Impairment Blindness.

[bib1245] McGlone F., Wessberg J., Olausson H. (2014). Discriminative and affective touch: sensing and feeling. Neuron.

[bib1250] McGurk H., Macdonald J. (1976). Hearing lips and seeing voices. Nature.

[bib1255] Meaux E., Gillet P., Bonnet-Brilhault F., Barthelemy C., Batty M. (2011). Atypical perception processing and facial emotion disorder in autism. Encephale.

[bib1260] Meltzoff A.N., Decety J. (2003). What imitation tells us about social cognition: a rapprochement between developmental psychology and cognitive neuroscience. Philos. Trans R. Soc. Lond. B Biol. Sci..

[bib1265] Menon V. (2011). Large-scale brain networks and psychopathology: a unifying triple network model. Trends Cogn. Sci..

[bib1270] Miall R.C., Weir D.J., Wolpert D.M., Stein J.F. (1993). Is the cerebellum a smith predictor?. J. Mot. Behav..

[bib1275] Miller Kuhaneck H., Britner P.A. (2013). A preliminary investigation of the relationship between sensory processing and social play in autism spectrum disorder. OTJR (Thorofare NJ).

[bib1280] Modahl C., Green L.A., Fein D., Morris M., Waterhouse L., Feinstein C., Levin H. (1998). Plasma oxytocin levels in autistic children. Biol. Psychiatry.

[bib1285] Mongillo E.A., Irwin J.R., Whalen D.H., Klaiman C., Carter A.S., Schultz R.T. (2008). Audiovisual processing in children with and without autism spectrum disorders. J. Autism Dev. Disord..

[bib1290] Moore J.K., Linthicum F.H. (2007). The human auditory system: a timeline of development. Int. J. Audiol..

[bib1295] Mosconi M.W., Mack P.B., McCarthy G., Pelphrey K.A. (2005). Taking an intentional stance on eye-gaze shifts: a functional neuroimaging study of social perception in children. Neuroimage.

[bib1300] Mottron L., Burack J.A., Burack, Charman, Yirmiya, Zelazo (2001). Enhanced perceptual functioning in the development of autism. The Development of Autism: Perspectives from Theory and Research.

[bib1305] Mottron L., Dawson M., Soulieres I., Hubert B., Burack J. (2006). Enhanced perceptual functioning in autism: an update, and eight principles of autistic perception. J. Autism Dev. Disord..

[bib1310] Mottron L., Peretz I., Ménard E. (2000). Local and global processing of music in high-functioning persons with autism: beyond central coherence?. J. Child Psychol. Psychiatry Allied Disciplines.

[bib1315] Mundy P., Crowson M. (1997). Joint attention and early social communication: implications for research on intervention with autism. J. Autism Dev. Disord..

[bib1320] Mundy P., Newell L. (2007). Attention, joint attention, and social cognition. Curr. Dir. Psychol. Sci..

[bib1325] Mundy P., Sullivan L., Mastergeorge A.M. (2009). A parallel and distributed-processing model of joint attention, social cognition and autism. Autism Res..

[bib1330] Muth A., Honekopp J., Falter C.M. (2014). Visuo-spatial performance in autism: a *meta*-analysis. J. Autism Dev. Disord..

[bib1335] Näätänen R., Broadbent D.E., James W. (1990). The role of attention in auditory information processing as revealed by event-related potentials and other brain measures of cognitive function. Behav. Brain Sci..

[bib1340] Nackaerts E., Wagemans J., Helsen W., Swinnen S.P., Wenderoth N., Alaerts K. (2012). Recognizing biological motion and emotions from point-light displays in autism spectrum disorders. PLoS One.

[bib1345] Nair A., Treiber J.M., Shukla D.K., Shih P., Müller R.A. (2013). Impaired thalamocortical connectivity in autism spectrum disorder: a study of functional and anatomical connectivity. Brain.

[bib1350] Narumoto J., Okada T., Sadato N., Fukui K., Yonekura Y. (2001). Attention to emotion modulates fMRI activity in human right superior temporal sulcus. Brain Res. Cogn. Brain Res..

[bib1355] Nebel M.B., Eloyan A., Nettles C.A., Sweeney K.L., Ament K., Ward R.E. (2016). Intrinsic visual-Motor synchrony correlates with social deficits in autism. Biol. Psychiatry.

[bib1360] Nomi J.S., Uddin L.Q. (2015). Face processing in autism spectrum disorders: from brain regions to brain networks. Neuropsychologia.

[bib1365] Novak L.R., Gitelman D.R., Schuyler B., Li W. (2015). Olfactory-visual integration facilitates perception of subthreshold negative emotion. Neuropsychologia.

[bib1370] O'Connor K. (2007). Brief report: impaired identification of discrepancies between expressive faces and voices in adults with Asperger’s syndrome. J. Autism Dev. Disord..

[bib1375] O'Connor K. (2012). Auditory processing in autism spectrum disorder: a review. Neurosci. Biobehav. Rev..

[bib1380] O’Riordan M., Passetti F. (2006). Discrimination in autism within different sensory modalities. J. Autism Dev. Disord..

[bib1385] O'Brien J., Spencer J., Girges C., Johnston A., Hill H. (2014). Impaired perception of facial motion in autism spectrum disorder. PLoS One.

[bib1390] Olausson H.L., Ekholm S., Strigo I., Worsley K., Vallbo Å., Bushnell M.C. (2002). Unmyelinated tactile afferents signal touch and project to insular cortex. Nat. Neurosci..

[bib1395] Olausson H., Wessberg J., McGlone F., Vallbo Å. (2010). The neurophysiology of unmyelinated tactile afferents. Neurosci. Biobehav. Rev..

[bib1400] Oram Cardy J.E., Flagg E.J., Roberts W., Roberts T.P.L. (2008). Auditory evoked fields predict language ability and impairment in children. Int. J. Psychophysiol..

[bib1405] Ozonoff S., Iosif A.M., Baguio F., Cook I.C., Hill M.M., Hutman T. (2010). A prospective study of the emergence of early behavioral signs of autism. J. Am. Acad. Child Adol. Psychiatry.

[bib1410] Palmer C.J., Paton B., Hohwy J., Enticott P.G. (2013). Movement under uncertainty: the effects of the rubber-hand illusion vary along the nonclinical autism spectrum. Neuropsychologia.

[bib1415] Palmer C.J., Seth A.K., Hohwy J. (2015). The felt presence of other minds: predictive processing, counterfactual predictions, and mentalising in autism. Conscious. Cogn..

[bib1420] Paton B., Hohwy J., Enticott P.G. (2012). The rubber hand illusion reveals proprioceptive and sensorimotor differences in autism spectrum disorders. J. Autism Dev. Disord..

[bib1425] Patriquin M.A., DeRamus T., Libero L.E., Laird A., Kana R.K. (2016). Neuroanatomical and neurofunctional markers of social cognition in autism spectrum disorder. Hum. Brain Mapp..

[bib1430] Paul R., Augustyn A., Klin A., Volkmar F.R. (2005). Perception and production of prosody by speakers with autism spectrum disorders. J. Autism Dev. Disord..

[bib1435] Paul R., Chawarska K., Fowler C., Cicchetti D., Volkmar F. (2007). Listen my children and you shall hear: auditory preferences in toddlers with autism spectrum disorders. J. Speech Lang. Hearing Res..

[bib1440] Pavlova M.A. (2012). Biological motion processing as a hallmark of social cognition. Cereb. Cortex.

[bib1445] Peiker I., David N., Schneider T.R. (2015). Perceptual integration deficits in autism spectrum disorders are associated with reduced interhemispheric gamma-band. Coherence.

[bib1450] Pellicano E., Burr D. (2012). When the world becomes ‘too real': a Bayesian explanation of autistic perception. Trends Cogn. Sci..

[bib1455] Pellicano E., Maybery M., Durkin K., Maley A. (2006). Multiple cognitive capabilities/deficits in children with an autism spectrum disorder: weak central coherence and its relationship to theory of mind and executive control. Dev. Psychopathol..

[bib1460] Pelphrey K.A., Carter E.J. (2008). Brain mechanisms for social perception: lessons from autism and typical development. Ann. N. Y. Acad. Sci..

[bib1465] Pelphrey K.A., Carter E.J. (2010). Brain mechanisms underlying social perception deficits in autism. Hum. Behav. Learn. Dev..

[bib1470] Pelphrey K.A., Morris J.P., McCarthy G. (2004). Grasping the intentions of others: the perceived intentionality of an action influences activity in the superior temporal sulcus during social perception. J. Cogn. Neurosci..

[bib1475] Pelphrey K.A., Morris J.P., McCarthy G., Labar K.S. (2007). Perception of dynamic changes in facial affect and identity in autism. Soc. Cogn. Affect. Neurosci..

[bib1480] Pelphrey K.A., Viola R.J., McCarthy G. (2004). When strangers pass: processing of mutual and averted social gaze in the superior temporal sulcus. Psychol. Sci..

[bib1485] Perrone J.A. (2007). Sensation and perception. Psychology in Aotearoa/New Zealand.

[bib1490] Pierce K., Müller R.A., Ambrose J., Allen G., Courchesne E. (2001). Face processing occurs outside the fusiform face area in autism: evidence from functional MRI. Brain.

[bib1495] Piwek L., Pollick F., Petrini K. (2015). Audiovisual integration of emotional signals from others’ social interactions. Front. Psychol..

[bib1500] Polan H., Ward M. (1994). Role of the mother's touch in failure to thrive: a preliminary investigation. J. Am. Acad. Child Adolesc. Psychiatry.

[bib1505] Purhonen M., Kilpeläinen-Lees R., Valkonen-Korhonen M., Karhu J., Lehtonen J. (2004). Cerebral processing of mother’s voice compared to unfamiliar voice in 4-month-old infants. Int. J. Psychophysiol..

[bib1510] Puts N.A., Wodka E.L., Tommerdahl M., Mostofsky S.H., Edden R.A. (2014). Impaired tactile processing in children with autism spectrum disorder. J. Neurophysiol..

[bib1515] Quaia C., Lefèvre P., Optican L.M. (2012). Model of the Control of Saccades by Superior Colliculus and Cerebellum Model of the Control of Saccades by Superior Colliculus and Cerebellum.

[bib1520] Quattrocki E., Friston K. (2014). Autism: oxytocin and interoception. Neurosci. Biobehav. Rev..

[bib1525] Redcay E. (2008). The superior temporal sulcus performs a common function for social and speech perception: implications for the emergence of autism. Neurosci. Biobehav. Rev..

[bib1530] Reece C., Ebstein R., Cheng X., Ng T., Schirmer A. (2016). Maternal touch predicts social orienting in young children. Cognit. Dev..

[bib1535] Reichow B. (2012). Overview of *meta*-Analyses on early intensive behavioral intervention for young children with autism spectrum disorders. J. Autism Dev. Disord..

[bib1540] Ring H.A., Baron-Cohen S., Wheelwright S., Williams S.C., Brammer M., Andrew C., Bullmore E.T. (1999). Cerebral correlates of preserved cognitive skills in autism: a functional MRI study of embedded figures task performance. Brain.

[bib1545] Riquelme I., Hatem S., Montoya P. (2016). Abnormal pressure pain, touch sensitivity, proprioception, and manual dexterity in children with Autism Spectrum Disorders. Neural Plas..

[bib1550] Roberts T.P.L., Khan S.Y., Rey M., Monroe J.F., Cannon K., Blaskey L., Woldoff S., Qasmieh S., Gandal M., Schmidt G.L., Zarnow D.M., Levy S.E., Edgar J.C. (2010). MEG detection of delayed auditory evoked responses in autism spectrum disorders: towards an imaging biomarker for autism. Autism Res..

[bib1555] Robertson C.E., Thomas C., Kravitz D.J., Wallace G.L., Baron-Cohen S., Martin A., Baker C.I. (2014). Global motion perception deficits in autism are reflected as early as primary visual cortex. Brain.

[bib1560] Rogers S.J., Ozonoff S. (2005). Annotation: what do we know about sensory dysfunction in autism? A critical review of the empirical evidence. J. Child Psychol. Psychiatry.

[bib1565] Ronconi L., Molteni M., Casartelli L. (2016). Building blocks of others' understanding: a perspective shift in investigating social-Communicative deficit in autism. Front. Hum. Neurosci..

[bib1570] Rosenblum L.D. (2008). Speech perception as a multimodal phenomenon. Curr. Dir. Psychol. Sci..

[bib1575] Rubin K.H., Coplan R., Chen X., Bowker J., McDonald K.L. (2011). Peer relationships in childhood. Soc. Pers. Dev..

[bib1580] Russell-Smith S.N., Maybery M.T., Bayliss D.M., Sng A.A. (2012). Support for a link between the local processing bias and social deficits in autism: an investigation of embedded figures test performance in non-clinical individuals. J. Autism Dev. Disord..

[bib1585] Russo N., Foxe J.J., Brandwein A.B., Altschuler T., Gomes H., Molholm S. (2010). Multisensory processing in children with autism: high-density electrical mapping of auditory-somatosensory integration. Autism Research.

[bib1590] Russo N.M., Skoe E., Trommer B., Nicol T., Zecker S., Bradlow A. (2008). Deficient brainstem encoding of pitch in children with Autism Spectrum Disorders. Clinical Neurophysiology. Off. J. Int. Feder. Clin. Neurophysiol..

[bib1595] Russo N., Nicol T., Trommer B., Zecker S., Kraus N. (2009). Brainstem transcription of speech is disrupted in children with autism spectrum disorders. Dev. Sci..

[bib1600] Rutgers A.H., Bakermans-Kranenburg M.J., Ijzendoorn M.H., Berckelaer-Onnes I.A. (2004). Autism and attachment: a *meta*-analytic review. J. Child Psychol. Psychiatry.

[bib1605] Rutherford M.D., Clements K.A., Sekuler A.B. (2007). Differences in discrimination of eye and mouth displacement in autism spectrum disorders. Vision Res..

[bib1610] Rutherford M.D., Young G.S., Hepburn S., Rogers S.J. (2007). A longitudinal study of pretend play in autism. J. Autism Dev. Disord..

[bib1615] Sacrey L.A.R., Armstrong V.L., Bryson S.E., Zwaigenbaum L. (2014). Impairments to visual disengagement in autism spectrum disorder: a review of experimental studies from infancy to adulthood. Neurosci. Biobehav. Rev..

[bib1620] Saitovitch A., Popa T., Lemaitre H., Rechtman E., Lamy J.C., Grevent D. (2016). Tuning eye-Gaze perception by transitory STS inhibition. Cereb. Cortex.

[bib1625] Samson F., Hyde K.L., Bertone A., Soulieres I., Mendrek A., Ahad P⋯., Zeffiro T.A. (2011). Atypical processing of auditory temporal complexity in autistics. Neuropsychologia.

[bib1630] Scheele D., Kendrick K., Khouri C., Kretzer E., Schläpfer T., Stoffel-Wagner B., Güntürkün O., Maier W., Hurlemann R. (2014). An oxytocin-induced facilitation of neural and emotional responses to social touch correlates inversely with autism traits. Neuropsychopharmacology.

[bib1635] Schelinski S., Riedel P., von Kriegstein K. (2014). Visual abilities are important for auditory-only speech recognition: evidence from autism spectrum disorder. Neuropsychologia.

[bib1640] Scherf K.S., Behrmann M., Minshew N., Luna B. (2008). Atypical development of face and greeble recognition in autism. J. Child Psychol. Psychiatry.

[bib1645] Schmitz N., Rubia K., Van Amelsvoort T., Daly E., Smith A., Murphy D.G. (2008). Neural correlates of reward in autism. Br. J. Psychiatry.

[bib1650] Schuetze M., Park M.T.M., Cho I.Y., MacMaster F.P., Chakravarty M.M., Bray S.L. (2016). Morphological alterations in the thalamus, striatum, and pallidum in autism spectrum disorder. Neuropsychopharmacology.

[bib1655] Schultz R.T., Gauthier I., Klin A., Fulbright R.K., Anderson A.W., Volkmar F. (2000). Abnormal ventral temporal cortical activity during face discrimination among individuals with autism and Asperger syndrome. Archives of general Psychiatry.

[bib1660] Senju A., Yaguchi K., Tojo Y., Hasegawa T. (2003). Eye contact does not facilitate detection in children with autism. Cognition.

[bib1665] Shams L., Kamitani Y., Shimojo S. (2000). Illusions. What you see is what you hear. Nature.

[bib1670] Shapiro T., Sherman M., Calamari G., Koch D. (1987). Attachment in autism and other developmental disorders. J. Am. Acad. Child Adolesc. Psychiatry.

[bib1675] Sherman S.M. (2007). The thalamus is more than just a relay. Curr. Opin. Neurobiol..

[bib1680] Shih P., Keehn B., Oram J.K., Leyden K.M., Keown C.L., Muller R.A. (2011). Functional differentiation of posterior superior temporal sulcus in autism: a functional connectivity magnetic resonance imaging study. Biol. Psychiatry.

[bib1685] Sigman M., Mundy P., Sherman T., Ungerer J. (1986). Social interactions of autistic, mentally retarded and normal children and their caregivers. J. Child Psychol. Psychiatry.

[bib1690] Silva L.M., Schalock M., Gabrielsen K.R. (2015). About face: evaluating and managing tactile impairment at the time of Autism diagnosis. Autism Res. Treat..

[bib1695] Silva L., Schalock M. (2016). First skin biopsy reports in children with autism show loss of C-Tactile. J. Neurol. Disord..

[bib1700] Silverman L.B., Bennetto L., Campana E., Tanenhaus M.K. (2010). Speech-and-gesture integration in high functioning autism. Cognition.

[bib1705] Simion F., Regolin L., Bulf H. (2008). A predisposition for biological motion in the newborn baby. Proc. Natl. Acad. Sci..

[bib1710] Simmons D.R., Robertson A.E., McKay L.S., Toal E., McAleer P., Pollick F.E. (2009). Vision in autism spectrum disorders. Vision Res..

[bib1715] Skuse D.H., Lori A., Cubells J.F., Lee I., Conneely K.N., Puura K., Lehtimäkie T., Binder E., Young L.J. (2014). Common polymorphism in the oxytocin receptor gene (OXTR) is associated with human social recognition skills. Proc. Natl. Acad. Sci..

[bib1720] Smith E.G., Bennetto L. (2007). Audiovisual speech integration and lipreading in autism. J. Child Psychol. Psychiatry Allied Disciplines.

[bib1725] Smythies J. (1997). The functional neuroanatomy of awareness: with a focus on the role of various anatomical systems in the control of intermodal attention. Conscious. Cogn..

[bib1730] Sokolov E.N. (1963). Higher nervous functions: the orienting reflex. Annu. Rev. Physiol..

[bib1735] Song Y., Hakoda Y., Sanefuji W., Cheng C. (2015). Can they see it? the functional field of view is narrower in individuals with autism spectrum disorder. PLoS One.

[bib1740] Spencer J.V., O'Brien J.M. (2006). Visual form-processing deficits in autism. Perception.

[bib1745] Stein B.E., Stanford T.R. (2008). Multisensory integration: current issues from the perspective of the single neuron. Nat. Rev. Neurosci..

[bib1750] Stern D.N. (1985). The Interpersonal World of the Infant. A View from Psychoanalysis and Developmental Psychology.

[bib1755] Stevenson R.A., Segers M., Ferber S., Barense M.D., Wallace M.T. (2014). The impact of multisensory integration deficits on speech perception in children with autism spectrum disorders. Front. Psychol..

[bib1760] Stevenson R.A., Siemann J.K., Woynaroski T.G., Schneider B.C., Eberly H.E., Camarata S.M., Wallace M.T. (2014). Evidence for diminished multisensory integration in autism spectrum disorders. J. Autism Dev. Disord..

[bib1765] Stevenson R.A., VanDerKlok R.M., Pisoni D.B., James T.W. (2011). Discrete neural substrates underlie complementary audiovisual speech integration processes. Neuroimage.

[bib1770] Stevenson R.A., Zemtsov R.K., Wallace M.T. (2012). Individual differences in the multisensory temporal binding window predict susceptibility to audiovisual illusions. J. Exp. Psychol. Hum. Percept. Perform..

[bib1775] Stienen B.M.C., Tanaka A., de Gelder B. (2011). Emotional voice and emotional body postures influence each other independently of visual awareness. PLoS One.

[bib1780] Stone W.L., Hoffman E.L., Lewis S.E., Ousley O.Y. (1994). Early recognition of autism. Parental reports vs clinical observation. Arch. Pediatr. Adolesc. Med..

[bib1785] Stoodley C.J., Schmahmann J.D. (2009). Functional topography in the human cerebellum: a meta-analysis of neuroimaging studies. Neuroimage.

[bib1790] Strata P., Scelfo B., Sacchetti B. (2011). Involvement of cerebellum in emotional behavior. Physiol. Res..

[bib1795] Suarez M.A. (2012). Sensory processing in children with autism spectrum disorders and impact on functioning. Pediatr. Clin. North Am..

[bib1800] Sutherland R.J., Whishaw I.Q., Kolb B. (1988). Contributions of cingulate cortex to two forms of spatial learning and memory. J. Neurosci..

[bib1805] Suzuki Y., Critchley H., Rowe A., Howlin P., Murphy D. (2003). Impaired olfactory identification in Asperger's syndrome. J. Neuropsychiatry Clin. Neurosci..

[bib1810] Swettenham J., Baron-Cohen S., Charman T., Cox A., Baird G., Drew A., Wheelwright S. (1998). The frequency and distribution of spontaneous attention shifts between social and nonsocial stimuli in autistic, typically developing, and nonautistic developmentally delayed infants. J. Child Psychol. Psychiatry.

[bib1815] Swettenham J., Remington A., Laing K., Fletcher R., Coleman M., Gomez J.C. (2013). Perception of pointing from biological motion point-light displays in typically developing children and children with autism spectrum disorder. J. Autism Dev. Disord..

[bib1820] Tager-Flusberg H., Paul R., Lord C. (2005). Language and communication in autism. Handb. Autism Pervasive Dev. Disord..

[bib1825] Takarae Y., Minshew N.J., Luna B., Krisky C.M., Sweeney J.A. (2004). Pursuit eye movement deficits in autism. Brain.

[bib1830] Tamura R., Kitamura H., Endo T., Hasegawa N., Someya T. (2010). Reduced thalamic volume observed across different subgroups of autism spectrum disorders. Psychiatry Res..

[bib1835] Tanabe H.C., Kosaka H., Saito D.N., Koike T., Hayashi M.J., Izuma K., Sadato N. (2012). Hard to tune in: neural mechanisms of live face-to-face interaction with high-functioning autistic spectrum disorder. Front. Hum. Neurosci..

[bib1840] Tannan V., Holden J.K., Zhang Z., Baranek G.T., Tommerdahl M.A. (2008). Perceptual metrics of individuals with autism provide evidence for disinhibition. Autism Res..

[bib1845] Tavassoli T., Baron-Cohen S. (2012). Taste identification in adults with autism spectrum conditions. J. Autism Dev. Disord..

[bib1850] Tavassoli T., Bellesheim K., Siper P.M., Wang A.T., Halpern D., Gorenstein M., Grodberg D., Kolevzon A., Buxbaum J.D. (2016). Measuring sensory reactivity in autism spectrum disorder: application and simplification of a clinician-administered sensory observation scale. J. Autism Dev. Disord..

[bib1855] Taylor N., Isaac C., Milne E. (2010). A comparison of the development of audiovisual integration in children with autism spectrum disorders and typically developing children. J. Autism Dev. Disord..

[bib1860] Teague S.J., Gray K.M., Tonge B.J., Newman L.K. (2017). Attachment in children with autism spectrum disorder: a systematic review. Res. Autism Spect. Disord..

[bib1865] Teder-Sälejärvi W.A., Pierce K.L., Courchesne E., Hillyard S.A. (2005). Auditory spatial localization and attention deficits in autistic adults. Cognit. Brain Res..

[bib1870] Teunisse J.P., Gelder B.D. (1994). Do autistics have a generalized face processing deficit?. Int. J. Neurosci..

[bib1875] Tomasello M. (1995). Joint attention as social cognition. Joint attention: its origins and role in development.

[bib1880] Tomchek S.D., Dunn W. (2007). Sensory processing in children with and without autism: a comparative study using the short sensory profile. Am. J. Occup. Ther..

[bib1885] Tsatsanis K.D., Rourke B.P., Klin A., Volkmar F.R., Cicchetti D., Schultz R.T. (2003). Reduced thalamic volume in high-functioning individuals with autism. Biol. Psychiatry.

[bib1890] Uddin L.Q., Menon V. (2009). The anterior insula in autism: under-connected and under examined. Neurosci. Biobehav. Rev..

[bib1895] Uljarević M., Lane A., Kelly A., Leekam S. (2016). Sensory subtypes and anxiety in older children and adolescents with autism spectrum disorder. Autism Res..

[bib1900] Uvnäs-Moberg K. (1998). Oxytocin may mediate the benefits of positive social interaction and emotions. Psychoneuroendocrinology.

[bib1905] Van Boxtel J.J., Lu H. (2013). A predictive coding perspective on autism spectrum disorders. Front. Psychology.

[bib1910] Van de Cruys S., de-Wit L., Evers K., Boets B., Wagemans J. (2012). 2013). Weak priors versus overfitting of predictions in autism: reply to Pellicano and Burr (TICS. Iperception.

[bib1915] Van Overwalle F., Baetens K., Mariën P., Vandekerckhove M. (2014). Social cognition and the cerebellum: a meta-analysis of over 350 fMRI studies. Neuroimage.

[bib1920] Vandenbroucke M.W.G., Scholte H.S., van Engeland H., Lamme V.A.F., Kemner C. (2008). A neural substrate for atypical low-level visual processing in autism spectrum disorder. Brain.

[bib1925] Vannetzel L., Chaby L., Cautru F., Cohen D., Plaza M. (2011). Neutral versus emotional human stimuli processing in children with pervasive developmental disorders not otherwise specified. Res. Autism Spectrum Disord..

[bib1930] Venkataraman A., Duncan J.S., Yang D.Y., Pelphrey K.A. (2015). An unbiased Bayesian approach to functional connectomics implicates social-communication networks in autism. Neuroimage Clin..

[bib1935] Villalobos M.E., Mizuno A., Dahl B.C., Kemmotsu N., Müller R.A. (2005). Reduced functional connectivity between V1 and inferior frontal cortex associated with visuomotor performance in autism. Neuroimage.

[bib1940] von der Luhe T., Manera V., Barisic I., Becchio C., Vogeley K., Schilbach L. (2016).

[bib1945] Vouloumanos A., Curtin S. (2014). Foundational tuning: how infants’ attention to speech predicts language development. Cognit. Sci..

[bib1950] Wallace G.L., Budgett J., Charlton R.A. (2016). Aging and autism spectrum disorder: evidence from the broad autism phenotype. Autism Res..

[bib1955] Wallace M.T., Stevenson R.A. (2014). The construct of the multisensory temporal binding window and its dysregulation in developmental disabilities. Neuropsychologia.

[bib1960] Wang A.T., Lee S.S., Sigman M., Dapretto M. (2006). Neural basis of irony comprehension in children with autism: the role of prosody and context. Brain.

[bib1965] Wass S.V., Jones E.J., Gliga T., Smith T.J., Charman T., Johnson M.H. (2015). Shorter spontaneous fixation durations in infants with later emerging autism. Sci. Rep..

[bib1970] Waterhouse L., Fein D., Modahl C. (1996). Neurofunctional mechanisms in autism. Psychol. Rev..

[bib1975] Weigelt S., Koldewyn K., Kanwisher N. (2012). Face identity recognition in autism spectrum disorders: a review of behavioral studies. Neurosci. Biobehav. Rev..

[bib1980] Weiss S.J., Wilson P., Hertenstein M.J., Campos R. (2000). The tactile context of a mother’s caregiving: implications for attachment of low birth weight infants. Infant Behav. Dev..

[bib1985] Weiss S.J., Wilson P., Seed M.S.J., Paul S.M. (2001). Early tactile experience of low birth weight children: links to later mental health and social adaptation. Infant Child Dev..

[bib1990] Wermter A.K., Kamp-Becker I., Hesse P., Schulte-Körne G., Strauch K., Remschmidt H. (2010). Evidence for the involvement of genetic variation in the oxytocin receptor gene (OXTR) in the etiology of autistic disorders on high-functioning level. Am. J. Med. Genet. B.

[bib1995] Wessberg J., Norrsell U. (1993). A system of unmyelinated afferents for innocuous mechanoreception in the human skin. Brain Res..

[bib2000] Whitehouse A.J.O., Bishop D.V.M. (2008). Do children with autism switch off to speech sounds? An investigation using event-related potentials. Dev. Sci..

[bib2005] Wiggins L., Robins D., Bakeman R., Adamson L. (2009). Brief report: sensory abnormalities as distinguishing symptoms of autism spectrum disorders in young children. J. Autism Dev. Disord..

[bib2010] Williams J.H.G., Massaro D.W., Peel N.J., Bosseler A., Suddendorf T. (2004). Visual-auditory integration during speech imitation in autism. Res. Dev. Disabil..

[bib2015] Williams J.H.G., Whiten A., Singh T. (2004). A systematic review of action imitation in autistic spectrum disorder. J. Autism Dev. Disord..

[bib2020] Wilson C.E., Brock J., Palermo R. (2010). Attention to social stimuli and facial identity recognition skills in autism spectrum disorder. J. Intellect. Disabil. Res..

[bib2025] Wolpert D.M., Miall R.C., Kawato M. (1998). Internal models in the cerebellum. Trends Cogn. Sci..

[bib2030] Wolpert D., Doya K., Kawato M. (2003). A unifying computational framework for motor control and social interaction. Philos. Trans. R Soc. Lond. B Biol. Sci..

[bib2035] Wu S., Jia M., Ruan Y., Liu J., Guo Y., Shuang M. (2005). Positive association of the oxytocin receptor gene (OXTR) with autism in the Chinese Han population. Biol. Psychiatry.

[bib2040] Xavier J., Vignaud V., Ruggiero R., Bodeau N., Cohen D., Chaby L. (2015). A multidimensional approach to the study of emotion recognition in autism spectrum disorders. Front. Psychol..

[bib2045] Yirmiya N., Gamliel I., Pilowsky T., Feldman R., Baron-Cohen S., Sigman M. (2006). The development of siblings of children with autism at 4 and 14 months: social engagement, communication, and cognition. J. Child Psychol. Psychiatry.

[bib2050] Yu L., Fan Y., Deng Z., Huang D., Wang S., Zhang Y. (2015). Pitch processing in tonal-Language-Speaking children with autism: an event-related potential study. J. Autism Dev. Disord..

[bib2055] Zalla T., Sav A.M., Leboyer M. (2009). Stimulus-reward association and reversal learning in individuals with Asperger Syndrome. Res. Autism Spect. Disord..

[bib2060] Zaytseva Y., Gutyrchik E., Bao Y., Pöppel E., Han S., Northoff G., Welker L., Meindl T., Blautzik J. (2014). Self processing in the brain: a paradigmatic fMRI case study with a professional singer. Brain Cogn..

[bib2065] Zou L.Q., Yang Z.Y., Wang Y., Lui S.S., Chen A.T., Cheung E.F., Chan R.C. (2016). What does the nose know? Olfactory function predicts social network size in human. Sci. Rep..

